# Female Anterior Cruciate Ligaments Exhibit a Muted Mechanobiological Response to Mechanical Loading

**DOI:** 10.1002/jor.70068

**Published:** 2025-10-15

**Authors:** Lauren Paschall, Maxwell Konnaris, Erdem Tabdanov, Aman Dhawan, Spencer E. Szczesny

**Affiliations:** ^1^ Department of Biomedical Engineering The Pennsylvania State University University Park Pennsylvania USA; ^2^ Pediatric Oncology Branch, Center for Cancer Research, The National Institutes of Health National Cancer Institute Bethesda Maryland USA; ^3^ Department of Statistics The Pennsylvania State University University Park Pennsylvania USA; ^4^ Department of Orthopaedics and Rehabilitation The Pennsylvania State University Hershey Pennsylvania USA; ^5^ Department of Pharmacology The Pennsylvania State University Hershey Pennsylvania USA

**Keywords:** anterior cruciate ligament, estrogen, mechanical loading, mechanobiology, tissue remodeling

## Abstract

Female athletes are significantly more likely to tear their anterior cruciate ligament (ACL) compared to their male counterparts. While there are several potential reasons for this, previous data from our lab demonstrated that female ACL explants have an impaired remodeling response to loading, which may prevent the repair of fatigue damage and lead to increased ACL rupture. The objective of this study was to identify the biological mechanisms driving the impaired remodeling of female ACLs to cyclic loading, including the role of estrogen. ACLs were harvested from male and female New Zealand white rabbits and cyclically loaded in a tensile bioreactor, followed by bulk RNA‐sequencing. Additional ACL explants treated with or without estradiol were analyzed using RT‐qPCR to determine the regulatory effect of estrogen on markers for tissue remodeling and inflammatory cytokines with cyclic loading. We found that female ACLs exhibited significantly fewer differentially expressed genes in response to loading compared to male ACLs. Additionally, multiple mechanotransduction pathways were enriched with loading only in the male ACLs. While a few estrogen‐related pathways were enriched in both male and female ACLs with loading, the expression of tissue remodeling markers was not different between estrogen treatment and vehicle control. Together, our findings highlight specific mechanotransduction pathways that may be responsible for the muted biological response of female ACLs to load, which provides a potential explanation for the increased rate of ACL tears in women.

## 1 Introduction

While anterior cruciate ligament (ACL) tears are common in general during athletic activity [1], female athletes are 2–8 times more likely to tear their ACLs compared to males [2, 3, 4]. Potential reasons for this include differences in anatomy, sex hormones, body size, and neuromuscular control [5, 6, 7]. However, women may also be more prone to ACL injury because of sex‐specific ACL remodeling in response to fatigue loading. Recent studies suggest that ACL injuries occur from fatigue failure and accumulation of tissue damage in the ACL [8, 9, 10, 11, 12, 13]. Previous data from our lab demonstrated that female rabbit ACL explants have an impaired remodeling response to load [14]. Specifically, we found that female ACLs fail to increase anabolic gene expression with loading and have increased catabolic gene expression compared to male ACL explants, which may prevent the repair of ACL fatigue damage and may explain the increased rate of ACL tears in women. However, the cause of this sex difference in ACL mechanobiology is unknown.

Differences in sex hormones (e.g., estrogen) are one obvious potential reason for the sex‐dependent ACL response to loading. Estrogen receptors (ERs) are present in both male and female ACL fibroblasts [15]. Additionally, estrogen influences multiple genes responsible for soft tissue remodeling [16, 17, 18, 19, 20, 21, 22, 23]; however, the magnitude and directionality of this effect on the ACL are inconsistent [16, 18, 19, 24, 25, 26, 27, 28, 29, 30, 31]. Despite these discrepancies, clinical data demonstrate that there are substantial changes in knee laxity during the menstrual cycle (average increase of 3 mm) that are correlated with estradiol concentrations [32]. Given that a 1.3 mm asymmetry in knee laxity is associated with a fourfold increase in ACL injury risk [33], these hormonal changes in knee laxity may help explain the increased ACL injury risk in women. Furthermore, changes in knee laxity can be attenuated through oral contraceptives that suppress spikes in estrogen levels [34, 35]. Together, these data suggest that estrogen has a direct effect on ACL tissue mechanics and may contribute to ACL rupture.

Previous data also suggest that estrogen regulates the response of cells to mechanical loading. A prior study on ACL fibroblasts found that mechanical loading completely reverses the effect of estrogen on gene expression [18]. However, the mechanisms underlying this interplay between estrogen and mechanical loading are unknown. In other tissues, ER signaling has been shown to interact with multiple mechanotransduction pathways (i.e., ERK, YAP/TAZ) [36, 37, 38, 39, 40, 41, 42, 43, 44]. However, the directionality of this effect is inconsistent. Specifically, studies on cancer cells show that ER signaling both increases [42, 43, 44] and decreases [39, 40, 41] YAP activation. While previous studies demonstrate that ER signaling regulates cellular mechanotransduction, it is not possible to predict the effect of estrogen on ACL fibroblast mechanotransduction from prior data alone.

This study builds upon our prior findings in which we demonstrated that female ACLs have an impaired remodeling response to mechanical loading compared to male ACLs [14]. While those findings exhibited sex‐based differences in ACL mechanobiology, the underlying signaling mechanisms remain unclear. Therefore, the objective of this study was to identify potential biological mechanisms driving the impaired remodeling response of female ACLs. We conducted bulk RNA‐sequencing on cyclically loaded male and female rabbit ACL explants to determine the innate sex differences in ACL mechanobiology in the native tissue environment. Additionally, we investigated the effect of estrogen treatment on the expression of remodeling genes in male and female loaded ACL explants. Our overall hypothesis was that female ACLs would exhibit an impaired mechanobiological response to load (increased catabolism, decreased anabolism), and this response would be influenced by estrogen. Specifically, we hypothesized that female ACLs would have altered mechanotransduction (suppression of ERK and YAP) as well as increased ER activity in response to load compared to males. We also hypothesized that estrogen treatment would increase catabolic and inflammatory gene expression in both male and female ACL explants in response to load.

## 2 Materials and Methods

### 2.1 ACL Harvest

A total of 33 male and female white New Zealand rabbits (2.8–3.2 kg, 14–16 weeks old, Charles River) were euthanized (Supporting Information S1: Table 1), and ACLs were isolated under sterile conditions from a study approved by the Institutional Animal Care and Use Committee at Penn State University. This age range coincides with the onset of sex differences in serum estrogen levels in rabbits [45]. ACLs were harvested following a previously published protocol [14]. Briefly, all surrounding tissues were removed, and the femur and tibia were cut into bone blocks to grip during mechanical loading. Cross‐sectional area was determined by assuming an elliptical cross‐section, and major and minor diameters were determined using calipers.

### 2.2 Mechanical Loading

ACLs were placed in a custom tensile bioreactor [46] with culture media (low‐glucose DMEM, 2% penicillin‐streptomycin, 5% FBS, 25 mm HEPES, 4 mm GlutaMAX, 1 mm 2‐phospho‐L‐ascorbic acid trisodium) and kept at 37°C and 5% CO_2_. A subset of the ACLs was untreated to determine the innate mechanobiology of male and female ACL explants. To investigate the effect of estrogen, ACLs were treated with 10 nM 17β‐estradiol (E2) (Sigma) or ethanol (0.1% v/v) as a vehicle control. The E2 concentration was based on a previous study investigating the role of estrogen and cyclic loading on isolated ACL fibroblasts [18]. Since underloading is detrimental to tendon/ligament homeostasis [47, 48], a 0.1 MPa static load was applied for 18 and 20 h to acclimate the untreated and treated (vehicle/estrogen) ACLs to culture conditions, respectively. The 18‐h timepoint was chosen to replicate previous work that found differences in the expression of remodeling genes with cyclic loading [14] for the RNA‐sequencing, and the 20‐h timepoint was chosen to be more consistent with prior studies of estrogen treatment on ACL mechanobiology [18]. The bioreactor then cyclically loaded the samples to 4 MPa at 0.5 Hz for 8 h. This stress level was chosen since it represents the stress that exhibited the largest gene expression differences between male and female ACLs [14]. Control samples were maintained under a 0.1 MPa static load for the same duration. Previous data from our lab demonstrated that these loading parameters maintain cell viability in the explants [14, 46].

### 2.3 RNA Extraction

Immediately following loading, samples were removed, and RNA was extracted following a previously published protocol [14]. Briefly, ACLs were excised from the bone blocks and were rinsed with ice‐cold RNase‐free water and flash frozen in liquid nitrogen. The tissue was pulverized (6775 Freezer/Mill, SPEX) and total RNA was extracted with RNeasy minicolumns (RNeasy Fibrous Tissue Kit, Qiagen). RNA concentration was quantified using a Qubit 4 Fluorometer (Thermo Fisher), and RNA integrity numbers (RINs) were determined using a TapeStation (Agilent).

### 2.4 RNA‐Sequencing

Samples that passed quality control (RIN > 6, total RNA > 250 ng) were sent to GENEWIZ (South Plainfield, NJ) for bulk RNA‐Sequencing (Supporting Information S1: Table 1). cDNA libraries were prepared using the NEBNext Ultra II RNA Library Prep for Illumina using the manufacturer's instructions (NEB, Ipswich). The sequencing libraries were validated on the Agilent TapeStation (Agilent Technologies) and quantified using a Qubit 3.0 Fluorometer (Invitrogen) as well as qPCR (KAPA Biosystems). The sequencing libraries were clustered on a lane of a NovaSeq. 6000 S4 flow cell. The samples were sequenced with an average of 32 million reads per sample using a 2 × 150 bp paired‐end configuration. Image analysis and base calling were conducted by the Control software. Raw sequence data (.bcl files) generated by the sequencer were converted into fastq files and de‐multiplexed using Illumina's bcl2fastq 2.17 software. One mismatch was allowed for index sequence identification. Sequenced reads were trimmed to remove possible adapter sequences and nucleotides with poor quality using Trimmomatic v.0.36. The trimmed reads were mapped to the OryCun2.0 reference genome available on ENSEMBL using the STAR aligner v.2.5.2b. Unique gene hit counts were calculated by using FeatureCount from the Subread package v.1.5.2.

### 2.5 Differential Gene Expression Analysis

**Figure 1:**
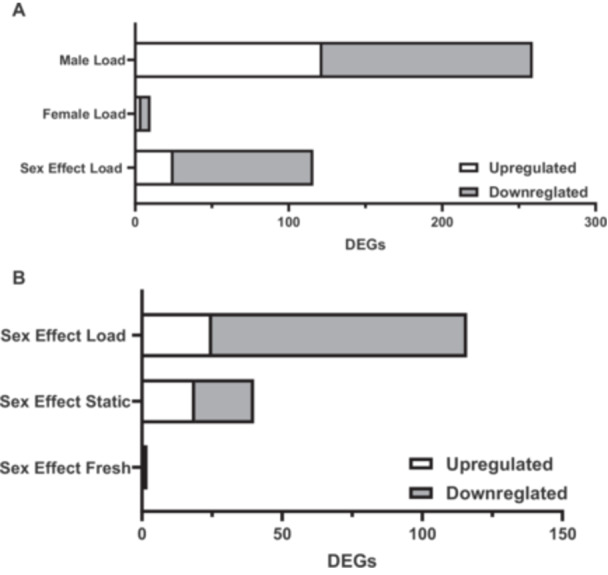
Differentially expressed genes. (A) Response of male and female ACLs in response to cyclic loading and comparing the response between sexes (sex effect load) (*n* = 3 males, *n* = 2 females). Male and female loads are relative to their sex‐matched static load. Sex effect load is comparing the female ACL response to load to the male ACL response to load. (B) Comparison of baseline gene expression differences of female ACLs relative to male ACLs (*n* = 3 static, *n* = 2 fresh). All DEGs had an absolute log2FC > 1 and an adjusted *p*‐value < 0.05.

Analysis and visualization of the bulk RNA‐sequencing was performed utilizing the NIH Integrated Analysis Platform (NIDAP) bulk RNA‐sequencing pipeline that utilizes R programs developed by a team of NCI bioinformaticians (Plantair Technologies, Denver, Colorado). Raw hit counts were uploaded, and quality control measures were performed using principal component analysis and supervised hierarchical clustering to determine intergroup relationships and identify outliers. A female‐loaded sample (FL3) was identified as an outlier and removed (Supporting Information S2: Figure 1). After outlier removal and normalization, differentially expressed gene (DEG) analysis was conducted utilizing the Limma Voom R package. Five comparisons were made to compare the response of each sex to load (female load, male load) and the relationships between sex in the fresh (sex effect fresh), static (sex effect static), and loaded (sex effect load) samples. The static control and male samples were used as the baseline for the comparisons. A moderated *t*‐statistic was used to determine *p*‐values, and a Benjamini–Hochberg false discovery rate was used to obtain the adjusted *p*‐values. Genes with an adjusted *p*‐value < 0.05 and |log2 fold change| > 1 were identified as DEGs. A list of DEGs was extracted from the model for each of the five comparisons.

### 2.6 Pathway Analysis and Upstream Regulator Analysis

After differential gene expression analysis, all DEGs for each comparison were uploaded into Ingenuity Pathway Analysis (IPA, Qiagen, Version Q4 2024). Significantly enriched pathways were determined with a −log(*p*‐value) > 1.3. Additionally, upstream regulator analysis was performed to determine potential regulators that could explain the observed changes in gene expression. To identify significantly activated or inhibited pathways or upstream regulators, a *z*‐score was calculated in IPA for each pathway or upstream regulator. A positive *z*‐score suggests inhibition, while a negative *z*‐score suggests activation. The magnitude of the *z*‐score reflects the confidence of the predicted activation or inhibition state, with |*z*‐scores | > 2 considered a strong prediction and |*z*‐scores| > 1 considered a moderate prediction.

### 2.7 Reverse Transcription Quantitative Polymerase Chain Reaction (RT‐qPCR)

The RNA extracted from each sample was diluted to 1 ng/ul, and cDNA was synthesized (High‐Capacity cDNA Reverse Transcription Kit with RNase Inhibitor, Thermo Fisher). qPCR was performed using TaqMan probes (Supporting Information S1: Table 2) and a StepOne Plus Real‐Time PCR system to measure the expression of anabolic (C*OL1A1*, *COL1A2*, *LOX, COL3A1, TGFβ1, ACTA, TIMP1, TIMP3*), catabolic (*MMP1, MMP2, MMP10, MMP13*), and inflammatory (*IL‐1β, PTGS2*) genes. Additionally, ER target genes (*ESR1, GPER1, PGR*) were measured along with *GAPDH* as the reference gene. PCR efficiency and cycle number quantification (Cq) were obtained for each individual reaction using PCR‐Miner (version 4.0) [49]. Samples that exhibited undetectable fluorescence for a given gene were excluded from the analysis for that gene. An outlier test (ROUT) was conducted to determine if any of the reaction efficiencies for each gene were an outlier. After removal of outliers, a single efficiency value was calculated for each probe by averaging all the individual sample efficiencies. Gene expression was quantified using the delta‐delta Cq method, correcting for primer efficiencies [50]. The fold change for each sample was calculated as:

$$\mathrm{Fold}\;\mathrm{change}=E_{\text{target}}(-∆C_{q})/E_{\text{ref}}(-∆C_{q}),$$

$$∆C_{q}=(Cq_{\text{experimental sample}}-\text{mean}\left[Cq_{\text{control group}}\right]),$$

E=1+probe efficiency,

where the probe efficiencies are listed in Supporting Information S1: Table 2.

### 2.8 Statistical Analysis of RT‐qPCR Data

The fold change for each gene was log‐normalized, and Mann–Whitney tests were conducted on the normalized data to compare the gene expression between treatments and sex. To determine the effect of mechanical loading on treatment, the estrogen‐treated and ethanol vehicle control samples were compared to their treatment‐matched statically loaded control samples (for both males and females). To determine baseline treatment‐specific gene expression differences, the estrogen‐treated static control samples were compared to the vehicle control static control samples (for both males and females). To determine baseline sex‐specific gene expression differences, the female vehicle control samples were compared to the male vehicle control samples. Additionally, Mann–Whitney tests were used on the transformed data to determine the differential expression of each gene compared to the respective control condition. Given the numerous statistical comparisons, *p*‐values were corrected using a false discovery rate analysis within each gene category (anabolic, catabolic, inflammatory, and ER target genes) with a *Q*‐value of 5%. Statistical significance after corrections was set as *p* < 0.05, with statistical trends set at *p* < 0.10. All statistical analysis was performed using GraphPad Prism (version 10.3.1).

## 3 Results

### 3.1 Differential Gene Expression Analysis

In response to cyclic loading, male ACLs had 259 DEGs (122 upregulated, 137 downregulated) while female ACLs only had 10 DEGs (4 upregulated, 6 downregulated). When comparing the response to load between female and male ACLs (sex effect load), there are 116 DEGs (25 upregulated, 91 downregulated in female ACLs compared to males) (Figure 1A, Supporting Information S1: Tables 3–5). When looking at the top 20 DEGs in the male samples' response to load, 75% of the DEGs were downregulated. Similarly, for the sex effect load comparison, 70% of the DEGs were further downregulated in female ACLs in response to load compared to males (Table 1). These differences are despite the fact that there are minimal differences between female and male ACLs before loading. Specifically, there were only 40 DEGs (19 upregulated, 21 downregulated) in female static ACLs compared to male samples, and only 2 DEGs (1 upregulated, 1 downregulated) in female freshly harvested ACLs compared to males (Figure 1B, Supporting Information S1: Tables 6 and 7).

**Table 1:** List of top 20 DEGs in response to load in male ACLs (and the sex effect of load (female ACLs response to load relative to male ACLs response to load).

Male load			Sex effect load		
Gene ID	Log2FC	*p*_adj_	Gene ID	Log2FC	*p*_adj_
ALK	−6.791	0.04	BARHL1	6.258	0.02
COX6	−6.299	0.01	SOSTDC1	−6.100	0.02
ENSOCUG00000013331	5.807	0.01	COL21A1	6.073	0.04
DSG2	−5.457	0.04	IL1RL1	−5.616	0.01
MASP1	−5.340	0.03	NALCN	−5.561	0.01
COL21A1	−5.169	0.02	CDO1	−5.527	0.02
NEK2	−5.097	0.02	GBP4	5.455	0.02
ENSOCUG00000023797	−5.060	0.02	CYTL1	−5.116	0.01
NPS	4.549	0.04	OLFM3	4.759	0.03
MANEAL	4.402	0.04	ANGPTL7	−4.747	0.02
IL1RL1	4.353	0.01	GALNT15	−4.625	0.02
IL2RA	−4.289	0.02	CADM4	4.375	0.05
RSPO1	−4.214	0.03	ENSOCUG00000029275	4.334	0.04
CAPN3	−4.209	0.03	ENSOCUG00000029534	−4.116	0.02
SPON1	−4.151	0.02	CCDC3	−3.965	0.01
KIF23	−4.130	0.03	MYOC	−3.838	0.02
PENK	−4.075	0.05	ADAMTSL3	−3.733	0.05
RASL12	−4.013	0.04	PRELP	−3.687	0.01
APLN	−4.004	0.04	NOVA1	−3.663	0.02
NALCN	3.958	0.01	MAMDC2	−3.595	0.01

Genes indicative of tissue remodeling (anabolic, catabolic, and inflammatory markers) [14] were searched in the list of DEGs for all five comparisons. In response to load, male ACLs downregulated catabolic gene *MMP2* with inflammatory marker *IL1β* trending toward downregulation, while female ACLs exhibited no differentially expressed remodeling genes (Table 2). When comparing across sex, there were no differentially expressed remodeling genes for any of the comparisons. (Table 2).

**Table 2:** Differential expression of ECM remodeling genes in all comparisons.

	Male load		Female load		Sex effect load		Sex effect static		Sex effect fresh	
Gene name	Log2FC	*p*_adj_	Log2FC	*p*_adj_	Log2FC	*p*_adj_	Log2FC	*p*_adj_	Log2FC	*p*_adj_
COL1A1	0.13	0.96	−0.86	0.50	−0.99	0.58	0.98	0.51	0.56	0.89
COL1A2	0.26	0.85	−0.91	0.36	−1.17	0.40	0.95	0.40	1.22	0.60
LOX	NA	NA	NA	NA	NA	NA	NA	NA	NA	NA
COL3A1	−0.04	0.98	−1.62	0.17	−1.58	0.28	0.65	0.62	1.19	0.60
TGFB1	0.06	0.92	−0.06	0.91	−0.12	0.85	0.10	0.86	−0.08	0.95
ACTA2	−2.40	0.11	−0.22	0.88	2.18	0.24	−0.72	0.59	−1.22	0.61
TIMP1	0.55	0.47	−0.54	0.40	−1.09	0.25	0.12	0.92	−0.62	0.59
TIMP3	NA	NA	NA	NA	NA	NA	NA	NA	NA	NA
MMP1	−2.27	0.11	0.96	0.49	3.23	0.12	−1.12	0.49	0.38	0.90
MMP2	−1.62	**0.03**	−0.27	0.66	1.36	0.14	−0.82	0.23	0.29	0.85
MMP10	NA	NA	NA	NA	NA	NA	NA	NA	NA	NA
MMP13	NA	NA	NA	NA	NA	NA	NA	NA	NA	NA
IL1B	−2.13	*0.06*	−0.53	0.56	1.60	0.25	−1.22	0.23	−0.65	0.94
PTGS2	NA	NA	NA	NA	NA	NA	NA	NA	NA	NA

*Note:* Bold text indicates statistical significance (*p* < 0.05) and italicized text indicates trends (*p* < 0.10). NAs indicate that the gene was not present in RNA‐sequencing data.

### 3.2 Differentially Expressed ER Target Genes

Previously reported ER target genes [51, 52] were searched in the list of DEGs for all five comparisons. In response to load, male ACLs upregulated *ESR1* and multiple genes trended towards downregulation (*IGFBP4, TPD52L1, SERPINE1*) while female ACLs exhibited no differentially expressed ER target genes (Table 3). When comparing the DEGs across sex, there were surprisingly no differentially expressed ER target genes for the freshly harvested or static samples. In response to load, *ESR1* trended towards downregulation in female‐loaded ACLs compared to male‐loaded ACLs (Table 3).

**Table 3:** Differential expression of estrogen receptor target genes in all comparisons.

	Male load		Female load		Sex effect load		Sex effect static		Sex effect fresh	
Gene name	Log2FC	*p*_adj_	Log2FC	*p*_adj_	Log2FC	*p*_adj_	Log2FC	*p*_adj_	Log2FC	*p*_adj_
ESR1	3.542	**0.02**	−0.662	0.61	−4.204	**0.05**	2.386	0.16	0.339	0.81
IGFBP4	−1.989	*0.10*	−0.750	0.49	1.238	0.42	−0.583	0.62	−1.360	0.52
TPD52L1	2.396	*0.10*	−1.047	0.61	−3.444	0.17	0.158	0.96	1.560	0.49
GPER1	−2.192	0.12	0.360	0.75	2.552	0.16	−0.620	0.59	−0.213	0.96
STC2	−1.870	0.35	1.350	0.37	3.219	0.20	−0.188	0.95	0.348	0.95
RBBP8	−0.212	0.70	0.677	0.16	0.889	0.17	−0.031	0.97	−0.097	0.97
SIAH2	0.944	0.18	−0.025	0.98	−0.968	0.29	0.587	0.41	1.010	0.40
ADCY9	0.249	0.70	−0.201	0.76	−0.450	0.56	−0.020	0.98	−0.661	0.45
SERPINE1	−1.830	*0.08*	0.259	0.82	2.089	0.15	−0.706	0.55	0.810	0.61
EPHA4	−1.150	0.29	−0.348	0.73	0.802	0.55	−0.059	0.97	−0.906	0.60

*Note:* Bold text indicates statistical significance (*p* < 0.05) and italicized text indicates trends (*p* < 0.10).

### 3.3 Pathway Analysis of ACLs Response to Cyclic Load

In response to cyclic loading, IPA found that male ACLs had 139 signaling pathways enriched, while female ACLs only had 31 enriched pathways (Supporting Information S1: Tables 8 and 9). When directly comparing across sex (sex effect load), there were 82 pathways enriched (Supporting Information S1: Table 10).

We then investigated which estrogen‐related and mechanotransduction pathways were enriched with loading. For estrogen, extra‐nuclear estrogen signaling was enriched in male ACLs in response to load and was trending towards inhibition (*z*‐score: −1), while ESR‐mediated signaling was enriched in female ACLs in response to load (Supporting Information S1: Tables 8 and 9).

For mechanotransduction, male ACLs had 15 pathways enriched in response to load (Table 4) with 8 pathways predicted to be downregulated and zero pathways predicted to be upregulated (Table 4). The female ACLs exhibited no enriched pathways. Additionally, there were 9 pathways enriched when directly comparing across sex (sex effect load). Prominent pathways of interest included integrin signaling, FAK signaling, MAPK signaling, PI3K/AKT signaling, Rho GTPase cycle, and G‐protein coupled receptor signaling. When comparing enriched pathways between male and female ACLs in response to load, five pathways were enriched in both male load and sex effect of load, which were generally predicted to be inhibited (Table 5). Interestingly, FAK signaling was predicted to be inhibited in the sex effect load comparison, indicating an increased inhibition of FAK in female ACLs compared to male ACLs in response to load.

**Table 4:** Mechanotransduction pathways enriched in response to load.

Male load		Sex effect load	
Pathway	*Z*‐score	Pathway	*Z*‐score
Integrin cell surface interactions	−2	FAK signaling	−2
G‐protein coupled receptor signaling	−2	Gap junction signaling	−1
Class A/1 (Rhodopsin‐like receptors)	−1	WNT/β‐catenin signaling	0
RHO GTPase cycle	−1	RHO GTPase cycle	0
Extra‐nuclear estrogen signaling	−1	Integrin signaling	NA
cAMP‐mediated signaling	−1	Actin nucleation by ARP‐WASP complex	NA
FAK signaling	−1	Integrin cell surface interactions	NA
WNT/SHH axonal guidance signaling pathway	−1	Signaling by MET	NA
RAF/MAP kinase cascade	0	PI3K/AKT signaling	NA
RHO GTPases activate CIT	NA		
Signaling by ALK	NA		
Signaling by NOTCH3	NA		
Signaling by PDGF	NA		
Signaling by MET	NA		
PI3K/AKT signaling	NA		

*Note:* Pathways with a adjusted *p*‐value < 0.05. Pathways with a *z*‐score > 0 are predicted to be activated, and *z*‐score < 0 are predicted to be inhibited. A prediction was unable to be made for pathways with an NA *Z*‐score.

**Table 5:** Mechanotransduction pathways enriched in both male ACL response to load and sex effect load.

Pathway	Male load *Z*‐score	Sex effect load *Z*‐score
FAK signaling	−1	−2
Integrin cell surface interactions	−2	NA
RHO GTPase cycle	−1	0
Signaling by MET	NA	NA
PI3K/AKT signaling	NA	NA

*Note:* Pathways with a adjusted *p*‐value < 0.05. Pathways with a *z*‐score > 0 are predicted to be activated, and *z*‐score < 0 are predicted to be inhibited. A prediction was unable to be made for pathways with an NA *Z*‐score.

Finally, we also investigated pathways associated with extracellular matrix (ECM) organization. Male ACLs had 8 ECM pathways enriched in response to load (Table 6), while female ACLs had no enriched pathways. Three pathways were enriched when directly comparing across sex (sex effect load). These included collagen synthesis and degradation, activation and inhibition of matrix metalloproteinases, and elastic fiber formation. Interestingly, most of these pathways were predicted to be inhibited in the male ACLs' response to load. When looking at what pathways are different between male ACLs and female ACLs in response to load, two pathways related to collagen synthesis were enriched in both the male load and sex effect of load comparisons (Table 7).

**Table 6:** Extracellular matrix pathways enriched in response to load.

Male load		Sex effect load	
Pathway	*Z*‐score	Pathway	*Z*‐score
Collagen degradation	−2	Elastic fiber formation	NA
Inhibition of matrix metalloproteases	2	Collagen biosynthesis and modifying enzymes	NA
Degradation of the extracellular matrix	−2	Collagen chain trimerization	NA
Extracellular matrix organization	−2		
Collagen biosynthesis and modifying enzymes	−2		
Collagen chain trimerization	−2		
Assembly of collagen fibrils and other multimeric structures	NA		
Activation of matrix metalloproteinases	NA		

*Note:* Pathways with an adjusted *p*‐value < 0.05. Pathways with a *z*‐score > 0 are predicted to be activated, and *z*‐score < 0 are predicted to be inhibited. A prediction was unable to be made for pathways with an NA *Z*‐score.

**Table 7:** Extracellular matrix pathways enriched in both male ACLs response to load and sex effect load.

Pathway	Male load *Z*‐score	Sex effect load *Z*‐score
Collagen biosynthesis and modifying enzymes	−2	NA
Collagen chain trimerization	−2	NA

*Note:* Pathways with a adjusted *p*‐value < 0.05. Pathways with a *z*‐score > 0 are predicted to be activated, and *z*‐score < 0 are predicted to be inhibited. A prediction was unable to be made for pathways with an NA *Z*‐score.

### 3.4 Upstream Regulator Analysis of ACLs Response to Cyclic Load

IPA upstream regulator analysis identified several key regulators of male ACL response to load (Table 8), including multiple regulators related to sex hormones (beta‐estradiol, ERBB2, dihydrotestosterone, AR, ESR1), growth factors (TGFβ1, VEGF, IGF1) and inflammatory cytokines (TNF, LPS, IL1β, prostaglandin E2), with the majority of these regulators predicted to be inhibited. Only DMPK was predicted to be a regulator of the female ACLs' response to load (Table 8). In the sex effect load, AR and TGFβ1 were predicted to be inhibited, while TNF was predicted to be activated.

**Table 8:** List of top 20 predicted upstream regulators of response to load in female and male ACLs (relative to sex‐matched static control) and the sex effect of load (female ACL response to load relative to male ACL response to load).

Male load		Female load		Sex effect load	
Upstream regulator	Activation *z*‐score	Upstream regulator	Activation *z*‐score	Upstream regulator	Activation *z*‐score
TNF	−4	DMPK	NA	ARID1A	−2
TGFB1	−3			cycloheximide	−2
Lipopolysaccharide	−3			AR	−2
VEGF	−3			dexamethasone	−2
CSF2	−3			anisomycin	−1
IL1B	−3			TGFB1	−1
AGT	−2			TNF	1
ESR1	−2			PLAU	1
IGF1	−2			SORL1	1
Demethasone	2			FAM3C	0
PEAR1	3			CDCA8	0
GUCY1B1	−1			PDGF‐BB (complex)	0
SOLR1	−1			LAMA4	0
Prostaglandin E2	−1			NR1I3	NA
ERBB2	−1			GNB2	NA
PTEN	1			UBE3D	NA
AR	0			BRMS1	NA
Immunoglobulin (complex)	0			sesaminol	NA
Beta‐estradiol	0			BCL9/CREBBP/EP300/CTNNB1/LEF/TCF (complex)	NA
Dihydrotestosterone	0			candesartan cilexetil	NA

*Note:* All regulators had an adjusted *p*‐value < 0.05. Regulators with a *z*‐score > 0 are predicted to be activated, and *z*‐score < 0 are predicted to be inhibited. A prediction was unable to be made for pathways with an NA *Z*‐score.

### 3.5 Effects of Estrogen Treatment on the Remodeling Response of ACLs to Cyclic Load

**Figure 2:**
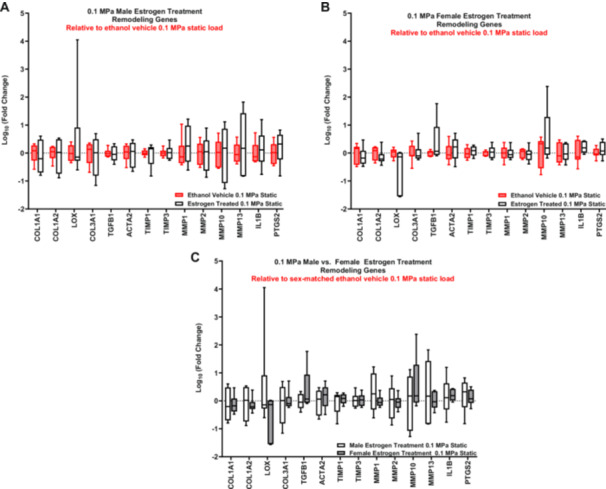
Basal remodeling gene expression response to estrogen treatment. RT‐qPCR analysis of statically loaded estrogen‐treated ACLs relative to their sex‐matched ethanol vehicle control (represented by the red box and whisker plots). (A) Statistically loaded male ACL response. (B) Statistically loaded female ACL response. (C) Comparison of statically loaded male and female ACL response to estrogen treatment (*n* = 6 for all male samples, *n* = 7 for female vehicle, *n* = 5–6 for female estrogen‐treated). Data represented as a box and whisker plot with the whiskers representing the min and max data.

When comparing the expression of remodeling genes between male and female statically loaded ACLs cultured without estrogen (vehicle control), there were multiple anabolic (*COL1A2, LOX, TGFβ1, TIMP3*), catabolic (*MMP1, MMP2, MMP13*), and inflammatory (*IL1β, PTGS2*) markers significantly upregulated in the female ACLs compared to males and *MMP1*0 was trending toward upregulation (Supporting Information S2: Figure 2A). However, there was no differential expression of any of the anabolic, catabolic, or inflammatory markers with estrogen treatment in the statically loaded male or female ACLs (Figure 2A,B). This was also true when comparing the response of estrogen treatment between male and female statically loaded ACLs (Figure 2C).

**Figure 3:**
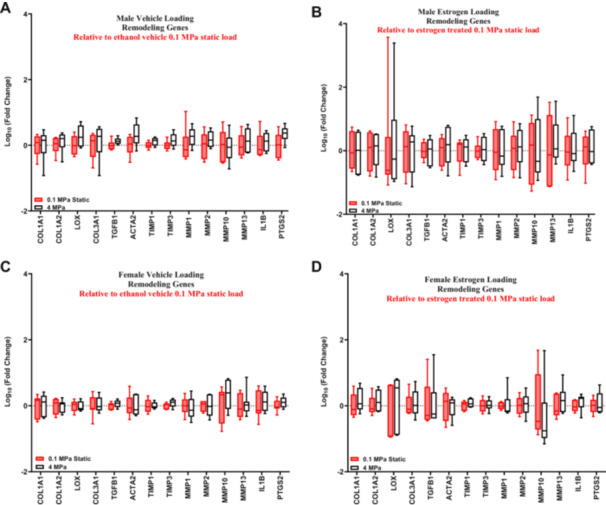
Effect of estrogen treatment on ACL remodeling in response to load. RT‐qPCR analysis of cyclically loaded male and female ACLs relative to their sex and treatment‐matched 0.1 MPa static load (represented by the red box and whisker plots). (A) Gene expression changes in vehicle control male ACLs. (B) Gene expression changes in estrogen‐treated male ACLs. (C) Gene expression changes in vehicle control female ACLs. (D) Gene expression changes in female estrogen‐treated ACLs (*n* = 6 for all male samples, *n* = 7 for female vehicle, *n* = 6 for female estrogen‐treated). Data represented as a box and whisker plot with the whiskers representing the min and max data.

No differential gene expression was observed with cyclical loading in the male ACLs compared to static controls with vehicle or estrogen treatment (Figure 3A,B). Direct comparisons between the vehicle control and estrogen‐treated male samples found that there was no effect of estrogen on gene expression (Supporting Information S2: Figure 3A). Consistently, the female cyclically loaded ACLs exhibited no differential gene expression compared to static controls with either vehicle or estrogen treatment (Figure 3C,D). Direct comparisons between vehicle and estrogen‐treated cyclically loaded female samples found no effect of estrogen on gene expression (Supporting Information S2: Figure 3B). Finally, direct comparison between the male and female cyclically loaded ACLs found that there were no sex differences in the vehicle or estrogen‐treated groups (Supporting Information S2: Figure 3C,D).

### 3.6 Effects of Estrogen Treatment on Estrogen Target Genes of ACLs to Cyclic Load

**Figure 4:**
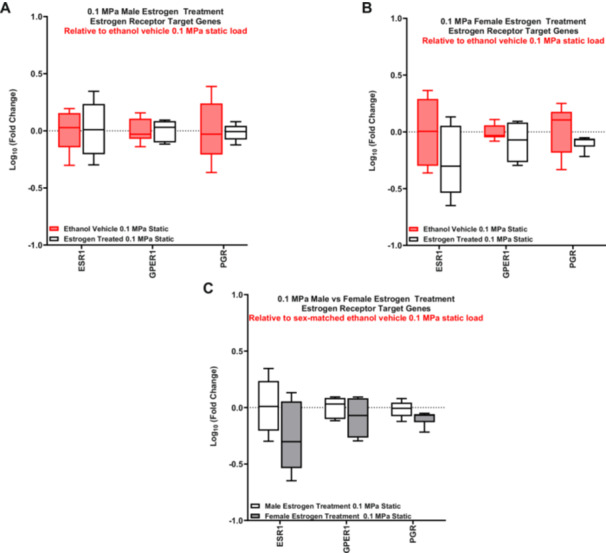
Basal estrogen receptor target genes response to estrogen treatment. RT‐qPCR analysis of statically loaded estrogen‐treated ACLs relative to their sex‐matched ethanol vehicle control (represented by the red box and whisker plots). (A) Statistically loaded male ACL response. (B) Statistically loaded female ACL response. (C) Comparison of statically loaded male and female ACLs response to estrogen treatment (*n* = 5 for male vehicle, *n* = 6 for male estrogen treated, *n* = 6–7 for female vehicle, *n* = 6 for female estrogen treated). Data represented as a box and whisker plot with the whiskers representing the min and max data.

In contrast to our RNA‐sequencing data (sex effect static), we found *GPER1* to be significantly upregulated in the statically loaded female ACLs compared to males without estrogen treatment (Supporting Information S2: Figure 2B). However, there was no differential expression of any of the ER genes in the statically loaded male or female ACLs in response to estrogen treatment (Figure 4A,B). Additionally, there were no differences in gene expression when comparing the response of estrogen treatment between male and female statically loaded ACLs (Figure 4C).

**Figure 5:**
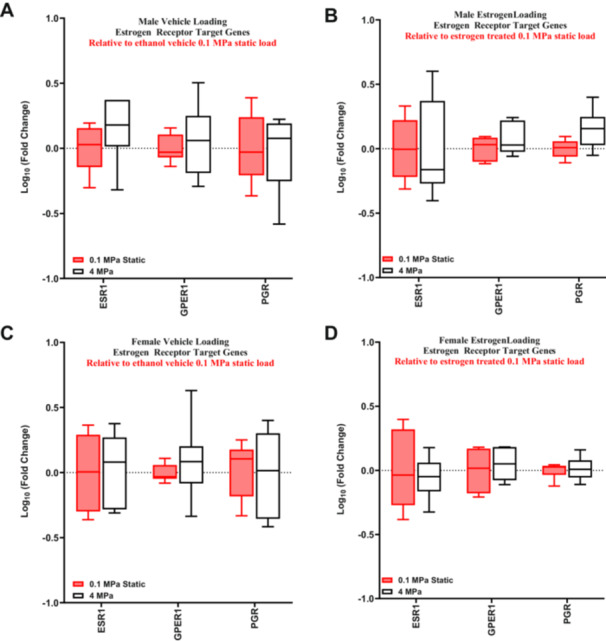
Effect of estrogen treatment on estrogen receptor target genes in response to load. RT‐qPCR analysis of cyclically loaded male and female ACLs relative to their sex and treatment‐matched 0.1 MPa static load. (A) Gene expression changes in vehicle control male ACLs. (B) Gene expression changes in estrogen‐treated male ACLs. (C) Gene expression changes in vehicle control female ACLs. (D) Gene expression changes in female estrogen‐treated ACLs (*n* = 6 for all male samples, *n* = 6–7 for female vehicle, *n* = 6 for female estrogen‐treated). Data represented as box and whisker plot with the whiskers representing the min and max data.

Consistent with our findings with the markers of tissue remodeling, there was no differential expression of the estrogen target genes with cyclic loading in the male (Figure 5A,B) or female samples (Figure 5C,D) with or without estrogen treatment. Similarly, there was no effect of estrogen (Supporting Information S2: Figure 4A,B) or sex (Supporting Information S2: Figure 4C,D) on the expression of estrogen target genes in response to loading.

## 4 Discussion

The objective of this study was to investigate potential mechanisms driving the impaired remodeling response of female ACLs in response to load. Consistent with our hypothesis, our RNA‐sequencing data demonstrated that female ACLs had a muted response to mechanical loading compared to male ACLs. Specifically, female ACLs had significantly fewer DEGs in response to load compared to male ACLs (Figure 1A). This is despite the fact that there were minimal differences in gene expression between freshly harvested female and male ACLs (Figure 1B). This suggests that the differences in the number of DEGs in response to load between male and female ACLs are due to differences in mechanobiology rather than basal biological differences. Furthermore, multiple mechanotransduction pathways were significantly enriched in male ACLs in response to load, while none were enriched in female ACLs (Table 4). Surprisingly, half of these mechanotransduction pathways were predicted to be downregulated in the male samples. However, a number of prominent mechanotransduction pathways, including MAPK (i.e., ERK) and PI3K/AKT, were enriched without likely being downregulated, suggesting that these pathways may be important for the mechanobiological response of male ACLs. Additionally, when comparing the response to load between male and female ACLs, FAK (focal adhesion kinase) signaling was predicted to be further inhibited with load in female ACLs compared to male ACLs (Tables 4 and 5). Therefore, FAK signaling may also help explain sex differences in ACL mechanobiology, given that FAK plays a prominent role in cell‐matrix mechanotransduction and regulates the mechanoresponse of tendon cells [53]. Together, our RNA‐sequencing data support the hypothesis that female ACLs have a muted mechanobiological response to load, potentially inhibiting their ability to repair tissue damage that predisposes the ACL to rupture.

Our secondary hypothesis was that the impaired mechanobiological response in female ACLs is due to estrogen signaling. Surprisingly, there were no basal differences in ER target genes between male and female ACLs (Table 3). This could be due to the fact that we did not measure estrogen concentrations to confirm that estrogen levels were higher in female rabbits at the time of euthanasia. While older rabbits closer to sexual maturity (16–24 weeks according to the supplier) may have produced a greater difference in gene expression, the age of the rabbits used in this study coincides with the onset of sex differences in serum estrogen levels [45]. Additionally, since rabbits are induced ovulators, female rabbits exhibit relatively stable serum estrogen concentrations over time [54]. These data suggest that the estrogen concentration should be consistently higher in the female ACL samples, suggesting that the chosen estrogen targets are not representative of estrogen signaling in rabbits. Downstream targets of estrogen activation are tissue and species‐specific [55, 56], and there are no data available on ER target genes in the ACL or rabbit, providing difficulties in validating ER activation. Additionally, we did not observe differential expression of the chosen ER targets in the female ACLs in response to load. However, the ESR‐mediated signaling pathway was enriched in the female ACL response to load (Supporting Information S1: Table 9). Additionally, the extra‐nuclear estrogen signaling was enriched and trending toward inhibition in the male ACL response to load (Supporting Information S1: Table 8). Furthermore, upstream regulator analysis revealed inhibition of ESR1 in the male ACL response to load (Table 8). Therefore, while rabbit‐specific markers of estrogen signaling may need to be identified, our RNA‐sequencing data suggest that estrogen‐related signaling may help explain sex differences in ACL mechanobiology.

To further investigate this, we cyclically loaded male and female ACLs in the presence of exogenous estradiol and measured gene expression of markers of ECM remodeling. Unfortunately, we were not able to replicate our previous work that utilized the identical loading protocol and identified a sex dependent response in these genes with loading [14]. Given that there was no effect of cyclic loading on our chosen remodeling genes, we were unable to evaluate the effect of estrogen (Figure 3, Supporting Information S2: Figure 3A,B). Power calculations were conducted to determine if this study is underpowered; however, the number of samples required to reach significance was infeasible (Supporting Information S1: Table 12). It is possible that the lack of effect with cyclic loading is due to a poor choice of gene markers for tissue remodeling. Specifically, while our RNA‐sequencing data found 259 DEGs with loading in male ACLs, only two of those genes were chosen as markers of tissue remodeling for our PCR experiments (*MMP2*, *IL1β*) (Table 2). However, when looking at the pathway analysis related to extracellular organization, there were only three pathways enriched when comparing the response to load between female and male ACLs (Table 6). Furthermore, only a few genes enriched in these pathways are relevant to remodeling of the bulk ECM (MMP2, MMP14, COL5A3), whereas the others are more relevant to the local pericellular matrix (ADAM10, ADAM12, COL4A1, COL4A2, LAMB1) (Supporting Information S1: Table 11). Together, this suggests that the mechanobiological difference between male and female ACLs may not involve bulk ECM remodeling. Still, the RNA‐sequencing data suggest that ACLs do have a sex‐dependent mechanobiological response, and future work is needed to identify the role of estrogen in driving this differential response.

It is also possible that the absence of an effect of estrogen on ACL explant gene expression was due to insufficient estradiol treatment. However, we used the same concentration (10^−8^ M) that increased expression of *COL1* and *COL3* in a previous study of isolated ACL fibroblasts [18]. Additionally, our chosen concentration is three orders of magnitude greater than serum estrogen concentrations found in rabbits (3.5 × 10^−11^ M) [25, 26]. It is possible that the ERs are already bound with endogenous estradiol in the female rabbits and that the male rabbits may have had a greater estradiol uptake. However, since there was no change in gene expression with treatment in the male explants, this suggests that there was no effect of estrogen on these genes, even if there was greater estradiol uptake in the male explants. Another possibility is that there was sufficient estradiol in the culture media to saturate estrogen signaling. While we did not use charcoal‐stripped FBS in our culture media, there is only approximately 2 pg/mL (< 10^−11^ M) of estradiol in 10% FBS, which was not sufficient to saturate the response of isolated ACL fibroblasts to estradiol [18]. A final possibility is that the effect of estrogen is no longer present at the end of our culture period (28 h), given that the effect of estrogen treatment on collagen gene expression decreased after 24 h [18].

Two final limitations to this study were that we did not evaluate cell viability in our explants and that our RNA‐sequencing sample size was small for the loaded female samples due to the removal of an outlier (*n* = 2) (Supporting Information S2: Figure 1). While prior experiments demonstrated that our protocols maintain cell viability in rabbit ACL explants [14], it is possible that the cell death may have affected our current results. Still, our RINs for the RNA‐sequencing data were high (7.5 ± 0.3) and our housekeeping genes were comparable between groups (Supporting Information S2: Figure 5), suggesting that there was comparable RNA quality and quantity between samples. Second, while it is possible that having a smaller sample size could reduce statistical power and lead to fewer DEGs, this is likely not the reason for the low number of DEGs in the female ACLs' response to load. Specifically, it is clear from the PCA plot of the samples included in the sequencing analysis (Supporting Information S2: Figure 1B) that the male‐loaded samples separated from the male static samples to a greater amount than the female samples, which is consistent with the fewer number of DEGs in the female ACLs in response to load.

In conclusion, our findings suggest that female ACLs have a muted mechanobiological response to load compared to male ACLs. This may be due to decreased activation of mechanotransduction pathways, including FAK, MAPK (i.e., ERK), and PI3K/AKT, as well as increased estrogen signaling in female ACLs in response to load. These data support the novel hypothesis that females exhibit an impaired mechanobiological response to load, making female athletes more susceptible to ACL tears. Future work could determine if these changes in gene expression lead to changes in tissue structure and mechanics by comparing the structural and mechanical changes that occur between male and female ACLs in response to load. This will enable a direct comparison of the male and female ACLs' ability to repair tissue damage. Future work is needed to understand better the role of estrogen signaling in ACL mechanobiology. Together, these data can provide insight into the ability of female ACLs to repair tissue damage and possible approaches for reducing the risk of ACL ruptures in females by modulating estrogen levels (e.g., contraceptive use).

## Author Contributions

Lauren Paschall carried out experiments and developed the protocols needed. Maxwell Konnaris provided input on the bioinformatic analysis. Lauren Paschall wrote the manuscript with support from all other authors. Erdem Tabdanov, Aman Dhawan, and Spencer E. Szczesny contributed to the interpretation of the results. Lauren Paschall and Spencer E. Szczesny conceived the original idea. All authors approve of the final submitted manuscript.

## Supporting information


**Supplementary Figure 1:** Principal component analysis. **Supplementary Figure 2:** Comparison of vehicle control female and male ACL gene expression at baseline. **Supplementary Figure 3:** Comparisons of the male and female ACLs remodeling response to load. **Supplementary Figure 4:** Comparisons of the male and female ACLs estrogen receptor target genes in response to load. **Supplementary Figure 5:** Comparison of housekeeping gene expression between groups.

**Supplementary Table 1:** Summary of sample sizes for each experimental condition. **Supplementary Table 2:** List of Taqman probes and their efficiencies. **Supplementary Table 3:** List of differentially expressed genes in male ACL response to load. **Supplementary Table 4:** List of differentially expressed genes in female ACL response to load. **Supplementary Table 5:** List of differentially expressed genes in sex effect load (female ACL response to load compared to male ACL response to load). **Supplementary Table 6:** List of differentially expressed genes in sex effect static (statically loaded female ACLs relative to male statically loaded ACLs). **Supplementary Table 7:** List of differentially expressed genes in sex effect fresh (freshly harvested female ACLs relative to male freshly harvested ACLs). **Supplementary Table 8:** List of significantly enriched pathways in male ACL response to load. Pathways with a z‐score > 0 are predicted to be activated and z‐score < 0 are predicted to be inhibited. A prediction was unable to be made for pathways with a NA z‐Score. **Supplementary Table 9:** List of significantly enriched pathways in female ACL response to load. A prediction was unable to be made for pathways with a NA z‐Score. **Supplementary Table 10:** List of significantly enriched pathways in sex effect load (female ACL response to load compared to male ACL response to load). Pathways with a z‐score > 0 are predicted to be activated and z‐score < 0 are predicted to be inhibited. A prediction was unable to be made for pathways with a NA z‐Score. **Supplementary Table 11:** List of DEGs present in pathways associated with ECM organization in male ACL response to load and sex effect load. **Supplementary Table 12:** Sample sizes required based on post‐hoc power calculations conducted on the PCR remodeling genes assuming a power > 0.8 and type I error of 0.05.

## References

[1] T. L. Sanders , H. Maradit Kremers , A. J. Bryan , et al., “Incidence of Anterior Cruciate Ligament Tears and Reconstruction: A 21‐Year Population‐Based Study,” American Journal of Sports Medicine 44, no. 6 (2016): 1502–1507. 26920430 10.1177/0363546516629944

[2] E. A. Arendt , J. Agel , and R. Dick , “Anterior Cruciate Ligament Injury Patterns Among Collegiate Men and Women,” Journal of Athletic Training 34, no. 2 (1999): 86–92. 16558564 PMC1322895

[3] C. C. Prodromos , Y. Han , J. Rogowski , B. Joyce , and K. Shi , “A Meta‐Analysis of the Incidence of Anterior Cruciate Ligament Tears as a Function of Gender, Sport, and a Knee Injury‐Reduction Regimen,” Arthroscopy: The Journal of Arthroscopic & Related Surgery 23, no. 12 (2007): 1320–1325.e6. 18063176 10.1016/j.arthro.2007.07.003

[4] A. M. Joseph , C. L. Collins , N. M. Henke , E. E. Yard , S. K. Fields , and R. D. Comstock , “A Multisport Epidemiologic Comparison of Anterior Cruciate Ligament Injuries in High School Athletics,” Journal of Athletic Training 48, no. 6 (2013): 810–817. 24143905 10.4085/1062-6050-48.6.03 PMC3867093

[5] S. J. Shultz and J. A. Fegley , “The Effect of Sex Hormones on Ligament Structure, Joint Stability and ACL Injury Risk,” in Sex Hormones, Exercise and Women: Scientific and Clinical Aspects, ed. A. C. Hackney (Springer International Publishing, 2023), 167–195, 10.1007/978-3-031-21881-1_8.

[6] M. L. Ireland , “The Female ACL: Why Is It More Prone to Injury?,” Orthopedic Clinics of North America 33, no. 4 (2002): 637–651. 12528906 10.1016/s0030-5898(02)00028-7

[7] T. E. Hewett , G. D. Myer , and K. R. Ford , “Anterior Cruciate Ligament Injuries in Female Athletes: Part 1, Mechanisms and Risk Factors,” American Journal of Sports Medicine 34, no. 2 (2006): 299–311. 16423913 10.1177/0363546505284183

[8] B. E. Loflin , T. Ahn , K. A. Colglazier , et al., “An Adolescent Murine In Vivo Anterior Cruciate Ligament Overuse Injury Model,” American Journal of Sports Medicine 51, no. 7 (2023): 1721–1732. 37092727 10.1177/03635465231165753 PMC10348391

[9] K. H. Putera , J. Kim , S. Y. Baek , et al., “Fatigue‐Driven Compliance Increase and Collagen Unravelling in Mechanically Tested Anterior Cruciate Ligament,” Communications Biology 6, no. 1 (2023): 564. 37237052 10.1038/s42003-023-04948-2 PMC10219950

[10] J. Chen , J. Kim , W. Shao , et al., “An Anterior Cruciate Ligament Failure Mechanism,” American Journal of Sports Medicine 47, no. 9 (2019): 2067–2076. 31307223 10.1177/0363546519854450 PMC6905051

[11] E. M. Wojtys , M. L. Beaulieu , and J. A. Ashton‐Miller , “New Perspectives on ACL Injury: On the Role of Repetitive Sub‐Maximal Knee Loading in Causing ACL Fatigue Failure,” Journal of Orthopaedic Research 34, no. 12 (2016): 2059–2068. 27653237 10.1002/jor.23441 PMC6362839

[12] L. H. Grodman , M. L. Beaulieu , J. A. Ashton‐Miller , and E. M. Wojtys , “Levels of ACL‐Straining Activities Increased in the Six Months Prior to Non‐Contact ACL Injury in a Retrospective Survey: Evidence Consistent With ACL Fatigue Failure,” Frontiers in Physiology 14 (2023): 1166980. 37215179 10.3389/fphys.2023.1166980 PMC10198379

[13] M. L. Beaulieu , M. G. DeClercq , N. T. Rietberg , et al., “The Anterior Cruciate Ligament Can Become Hypertrophied in Response to Mechanical Loading: A Magnetic Resonance Imaging Study in Elite Athletes,” American Journal of Sports Medicine 49, no. 9 (2021): 2371–2378. 34259598 10.1177/03635465211012354 PMC8561743

[14] L. Paschall , S. Carrozzi , E. Tabdanov , A. Dhawan , and S. E. Szczesny , “Cyclic Loading Induces Anabolic Gene Expression in ACLs in a Load‐Dependent and Sex‐Specific Manner,” Journal of Orthopaedic Research 42, no. 2 (2024): 267–276. 37602554 10.1002/jor.25677

[15] S. H. Liu , R. Al‐Shaikh , V. Panossian , et al., “Primary Immunolocalization of Estrogen and Progesterone Target Cells in the Human Anterior Cruciate Ligament,” Journal of Orthopaedic Research 14, no. 4 (1996): 526–533. 8764860 10.1002/jor.1100140405

[16] S. H. Liu , R. A. Al‐Shaikh , V. Panossian , G. A. M. Finerman , and J. M. Lane , “Estrogen Affects the Cellular Metabolism of the Anterior Cruciate Ligament. A Potential Explanation for Female Athletic Injury,” American Journal of Sports Medicine 25, no. 5 (1997): 704–709. 9302481 10.1177/036354659702500521

[17] H. Hama , T. Yamamuro , and T. Takeda , “Experimental Studies on Connective Tissue of the Capsular Ligament. Influences of Aging and Sex Hormones,” Acta Orthopaedica Scandinavica 47, no. 5 (1976): 473–479. 998180 10.3109/17453677608988723

[18] C.‐Y. Lee , X. Liu , C. L. Smith , et al., “The Combined Regulation of Estrogen and Cyclic Tension on Fibroblast Biosynthesis Derived From Anterior Cruciate Ligament,” Matrix Biology 23, no. 5 (2004): 323–329. 15464364 10.1016/j.matbio.2004.07.004

[19] C. A. Lee , A. Lee‐Barthel , L. Marquino , et al., “Estrogen Inhibits Lysyl Oxidase and Decreases Mechanical Function in Engineered Ligaments,” Journal of Applied Physiology (Bethesda, Md., 1985) 118, no. 10 (2015): 1250–1257. 25979936 10.1152/japplphysiol.00823.2014

[20] L. M. Wahl , R. J. Blandau , and R. C. Page , “Effect of Hormones on Collagen Metabolism and Collagenase Activity in the Pubic Symphysis Ligament of the Guinea Pig,” Endocrinology 100, no. 2 (1977): 571–579. 188632 10.1210/endo-100-2-571

[21] M. R. Rajabi , G. R. Dodge , S. Solomon , and A. R. Poole , “Immunochemical and Immunohistochemical Evidence of Estrogen‐Mediated Collagenolysis as a Mechanism of Cervical Dilatation in the Guinea Pig at Parturition,” Endocrinology 128, no. 1 (1991): 371–378. 1986929 10.1210/endo-128-1-371

[22] L. A. Salamonsen and D. E. Woolley , “Matrix Metalloproteinases in Normal Menstruation,” Human Reproduction 11, no. Suppl 2 (1996): 124–133. 8982754 10.1093/humrep/11.suppl_2.124

[23] J. Schneikert , H. Peterziel , P. A. Defossez , H. Klocker , Y. de Launoit , and A. C. B. Cato , “Androgen Receptor‐Ets Protein Interaction Is a Novel Mechanism for Steroid Hormone‐Mediated Down‐Modulation of Matrix Metalloproteinase Expression,” Journal of Biological Chemistry 271, no. 39 (1996): 23907–23913. 8798622 10.1074/jbc.271.39.23907

[24] J. Slauterbeck , C. Clevenger , W. Lundberg , and D. M. Burchfield , “Estrogen Level Alters the Failure Load of the Rabbit Anterior Cruciate Ligament,” Journal of Orthopaedic Research 17, no. 3 (1999): 405–408. 10376730 10.1002/jor.1100170316

[25] T. Komatsuda , T. Sugita , H. Sano , et al., “Does Estrogen Alter the Mechanical Properties of the Anterior Cruciate Ligament? An Experimental Study in Rabbits,” Acta Orthopaedica 77, no. 6 (2006): 973–980. 17260210 10.1080/17453670610013312

[26] K. Hattori , H. Sano , T. Komatsuda , Y. Saijo , T. Sugita , and E. Itoi , “Effect of Estrogen on Tissue Elasticity of the Ligament Proper in Rabbit Anterior Cruciate Ligament: Measurements Using Scanning Acoustic Microscopy,” Journal of Orthopaedic Science 15, no. 4 (2010): 584–588. 20721729 10.1007/s00776-010-1474-0

[27] S. M. Strickland , T. W. Belknap , S. A. Turner , T. M. Wright , and J. A. Hannafin , “Lack of Hormonal Influences on Mechanical Properties of Sheep Knee Ligaments,” American Journal of Sports Medicine 31, no. 2 (2003): 210–215. 12642254 10.1177/03635465030310020901

[28] F. A. Wentorf , K. Sudoh , C. Moses , E. A. Arendt , and C. S. Carlson , “The Effects of Estrogen on Material and Mechanical Properties of the Intra‐ and Extra‐Articular Knee Structures,” American Journal of Sports Medicine 34, no. 12 (2006): 1948–1952. 16861578 10.1177/0363546506290060

[29] S. J. Warden , L. K. Saxon , A. B. Castillo , and C. H. Turner , “Knee Ligament Mechanical Properties Are Not Influenced by Estrogen or Its Receptors,” American Journal of Physiology‐Endocrinology and Metabolism 290, no. 5 (2006): E1034–E1040. 16317027 10.1152/ajpendo.00367.2005

[30] W. D. Yu , S. H. Liu , J. D. Hatch , V. Panossian , and G. A. M. Finerman , “Effect of Estrogen on Cellular Metabolism of the Human Anterior Cruciate Ligament,” Clinical Orthopaedics and Related Research 366 (1999): 229–238. 10.1097/00003086-199909000-00030 10627740

[31] W. D. Yu , V. Panossian , J. D. Hatch , S. H. Liu , and G. A. M. Finerman , “Combined Effects of Estrogen and Progesterone on the Anterior Cruciate Ligament,” Clinical Orthopaedics and Related Research 383 (2001): 268–281. 10.1097/00003086-200102000-00031 11210964

[32] S. J. Shultz , S. E. Kirk , M. L. Johnson , T. C. Sander , and D. H. Perrin , “Relationship Between Sex Hormones and Anterior Knee Laxity Across the Menstrual Cycle,” Medicine & Science in Sports & Exercise 36, no. 7 (2004): 1165–1174. 15235320 10.1249/01.MSS.0000132270.43579.1A PMC1993893

[33] G. D. Myer , K. R. Ford , M. V. Paterno , T. G. Nick , and T. E. Hewett , “The Effects of Generalized Joint Laxity on Risk of Anterior Cruciate Ligament Injury in Young Female Athletes,” American Journal of Sports Medicine 36, no. 6 (2008): 1073–1080. 18326833 10.1177/0363546507313572 PMC3407802

[34] P. A. Martineau , F. Al‐Jassir , E. Lenczner , and M. L. Burman , “Effect of the Oral Contraceptive Pill on Ligamentous Laxity,” Clinical Journal of Sport Medicine 14, no. 5 (2004): 281–286. 15377967 10.1097/00042752-200409000-00006

[35] H. Lee , J. S. Petrofsky , and J. Yim , “Do Oral Contraceptives Alter Knee Ligament Damage With Heavy Exercise?,” Tohoku Journal of Experimental Medicine 237, no. 1 (2015): 51–56. 26346968 10.1620/tjem.237.51

[36] E. Garcia Dos Santos , M. Dieudonne , R. Pecquery , V. Le Moal , Y. Giudicelli , and D. Lacasa , “Rapid Nongenomic E2 Effects on p42/p44 MAPK, Activator Protein‐1, and cAMP Response Element Binding Protein in Rat White Adipocytes,” Endocrinology 143, no. 3 (2002): 930–940. 11861515 10.1210/endo.143.3.8678

[37] E. R. Levin , “Integration of the Extranuclear and Nuclear Actions of Estrogen,” Molecular Endocrinology 19, no. 8 (2005): 1951–1959. 15705661 10.1210/me.2004-0390 PMC1249516

[38] M. Marino , P. Galluzzo , and P. Ascenzi , “Estrogen Signaling Multiple Pathways to Impact Gene Transcription,” Current Genomics 7, no. 8 (2006): 497–508. 18369406 10.2174/138920206779315737 PMC2269003

[39] D. Lachowski , E. Cortes , C. Matellan , et al., “G Protein‐Coupled Estrogen Receptor Regulates Actin Cytoskeleton Dynamics to Impair Cell Polarization,” Frontiers in Cell and Developmental Biology 8 (2020): 592628. 33195261 10.3389/fcell.2020.592628 PMC7649801

[40] A. Rice , E. Cortes , D. Lachowski , et al., “GPER Activation Inhibits Cancer Cell Mechanotransduction and Basement Membrane Invasion via RhoA,” Cancers 12, no. 2 (2020): 289. 31991740 10.3390/cancers12020289 PMC7073197

[41] Y. Jeon , J. E. Yoo , H. Rhee , et al., “YAP Inactivation in Estrogen Receptor Alpha‐Positive Hepatocellular Carcinoma With Less Aggressive Behavior,” Experimental & Molecular Medicine 53, no. 6 (2021): 1055–1067. 34145394 10.1038/s12276-021-00639-2 PMC8257598

[42] Z. Wang , L. Sun , S. Liang , et al., “GPER Stabilizes F‐Actin Cytoskeleton and Activates TAZ via PLCβ‐PKC and Rho/ROCK‐LIMK‐Cofilin Pathway,” Biochemical and Biophysical Research Communications 516, no. 3 (2019): 976–982. 31277940 10.1016/j.bbrc.2019.06.132

[43] J. S. Muhammad , M. Guimei , M. N. Jayakumar , et al., “Estrogen‐Induced Hypomethylation and Overexpression of YAP1 Facilitate Breast Cancer Cell Growth and Survival,” Neoplasia 23, no. 1 (2021): 68–79. 33242831 10.1016/j.neo.2020.11.002 PMC7695929

[44] X. Zhou , S. Wang , Z. Wang , et al., “Estrogen Regulates Hippo Signaling via GPER in Breast Cancer,” Journal of Clinical Investigation 125, no. 5 (2015): 2123–2135. 25893606 10.1172/JCI79573 PMC4463207

[45] H. Takano , T. Aizawa , T. Irie , S. Kokubun , and E. Itoi , “Estrogen Deficiency Leads to Decrease in Chondrocyte Numbers in the Rabbit Growth Plate,” Journal of Orthopaedic Science 12, no. 4 (2007): 366–374. 17657557 10.1007/s00776-007-1145-y

[46] K. Pedaprolu and S. E. Szczesny , “A Novel, Open‐Source, Low‐Cost Bioreactor for Load‐Controlled Cyclic Loading of Tendon Explants,” Journal of Biomechanical Engineering 144, no. 8 (2022): 084505. 35147179 10.1115/1.4053795

[47] S. P. Arnoczky , T. Tian , M. Lavagnino , and K. Gardner , “Ex Vivo Static Tensile Loading Inhibits MMP‐1 Expression in Rat Tail Tendon Cells Through a Cytoskeletally Based Mechanotransduction Mechanism,” Journal of Orthopaedic Research 22, no. 2 (2004): 328–333. 15013092 10.1016/S0736-0266(03)00185-2

[48] T. Wang , Z. Lin , M. Ni , et al., “Cyclic Mechanical Stimulation Rescues Achilles Tendon From Degeneration in a Bioreactor System,” Journal of Orthopaedic Research 33, no. 12 (2015): 1888–1896. 26123799 10.1002/jor.22960

[49] S. Zhao and R. D. Fernald , “Comprehensive Algorithm for Quantitative Real‐Time Polymerase Chain Reaction,” Journal of Computational Biology 12, no. 8 (2005): 1047–1064. 16241897 10.1089/cmb.2005.12.1047 PMC2716216

[50] M. Pfaffl , “Relative Quantification,” in Real‐Time PCR, ed. M. Tevfik Dorak (Taylor and Francis, 2006), 63–82.

[51] C.‐Y. Lin , A. Ström , V. B. Vega , et al., “Discovery of Estrogen Receptor α Target Genes and Response Elements in Breast Tumor Cells,” Genome Biology 5, no. 9 (2004): R66. 15345050 10.1186/gb-2004-5-9-r66 PMC522873

[52] W. Verhaegh , H. van Ooijen , M. A. Inda , et al., “Selection of Personalized Patient Therapy Through the Use of Knowledge‐Based Computational Models That Identify Tumor‐Driving Signal Transduction Pathways,” Cancer Research 74, no. 11 (2014): 2936–2945. 24695361 10.1158/0008-5472.CAN-13-2515

[53] T. P. Leahy , S. S. Chenna , L. J. Soslowsky , and N. A. Dyment , “Focal Adhesion Kinase Regulates Tendon Cell Mechanoresponse and Physiological Tendon Development,” FASEB Journal 38, no. 17 (2024): e70050. 39259535 10.1096/fj.202400151R PMC11522781

[54] X. Chen , R. Jin , A. Yang , et al., “Behavioral and Physiological Differences in Female Rabbits at Different Stages of the Estrous Cycle,” Animals 13, no. 21 (2023): 3414. 37958169 10.3390/ani13213414 PMC10648029

[55] M. Kian Tee , I. Rogatsky , C. Tzagarakis‐Foster , et al., “Estradiol and Selective Estrogen Receptor Modulators Differentially Regulate Target Genes With Estrogen Receptors α and β,” Molecular Biology of the Cell 15, no. 3 (2004): 1262–1272. 14699072 10.1091/mbc.E03-06-0360 PMC363122

[56] D. Leitman , S. Paruthiyil , C. Yuan , et al., “Tissue‐Specific Regulation of Genes by Estrogen Receptors,” Seminars in Reproductive Medicine 30, no. 1 (2012): 14–22. 22271290 10.1055/s-0031-1299593

