# Supplementary material for: Female Anterior Cruciate Ligaments Exhibit a Muted Mechanobiological Response to Mechanical Loading
Source: J Orthop Res. 2025 Oct 15;43(12):2188–202. doi: 10.1002/jor.70068 (PMC12604462; doi:10.1002/jor.70068)
Supplement: Supplementary file 1 — Supplementary Figure 1: Principal component analysis. Supplementary Figure 2: Comparison of vehicle control female and male ACL gene expression at baseline. Supplementary Figure 3: Comparisons of the male and female ACLs remodeling response to load. Supplementary Figure 4: Comparisons of the male and female ACLs estrogen receptor target genes in response to load. Supplementary Figure 5: Comparison of housekeeping gene expression between groups. [file JOR-43-2188-s002.docx]

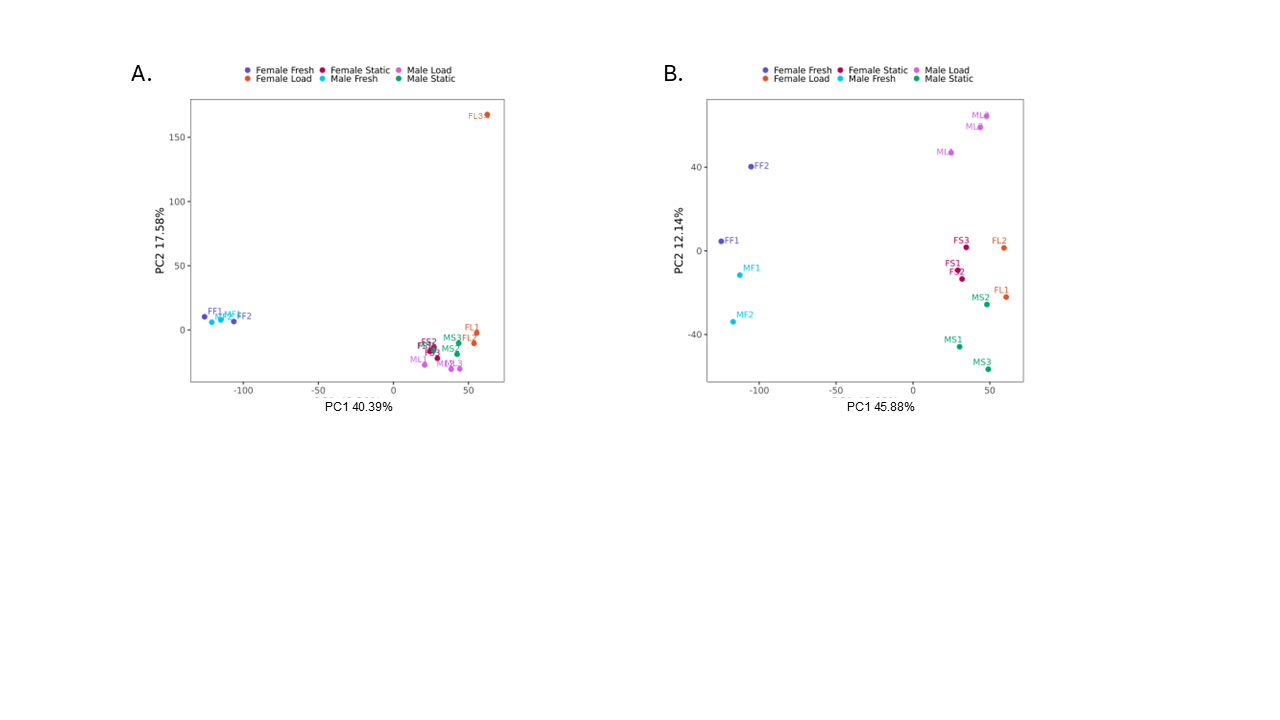


Supplementary Figure 1: **Principal component analysis.** Visual distribution of samples based on two principal components. Each point represents an individual sample, labeled with the sample number. (A) Principal component analysis without the outlier removal (FL3). (B) Principal component analysis with the outlier removal.


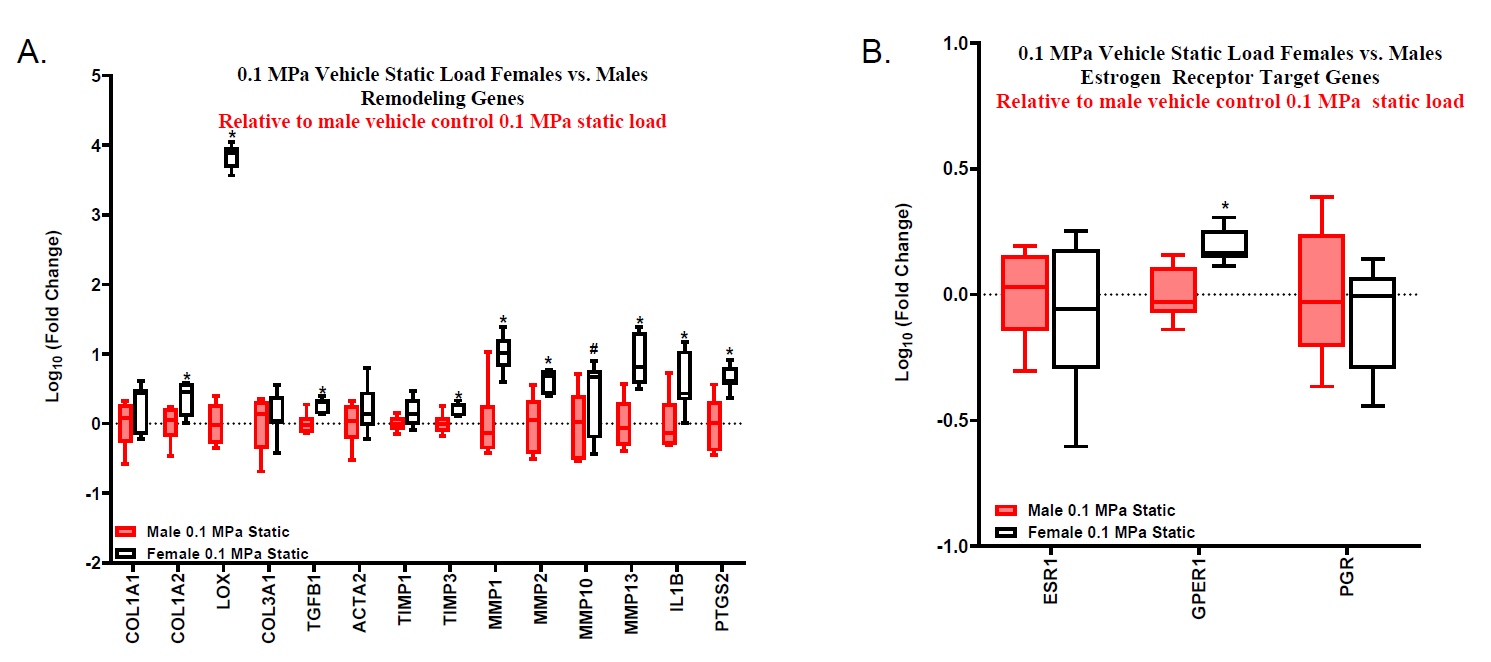


Supplementary Figure 2: **Comparison of vehicle control female and male ACL gene expression at baseline.** RT-qPCR analysis of statically loaded female samples relative to male statically loaded samples. (A) Remodeling genes. (B) Estrogen receptor target genes (n=6 for male samples and n=7 for female samples for remodeling genes, n=6 for male samples and n=6 - 7 for female samples for estrogen receptor target genes) *p<0.05, #p<0.1. Data represented as box and whiskers plot with the whiskers representing the min and max data.


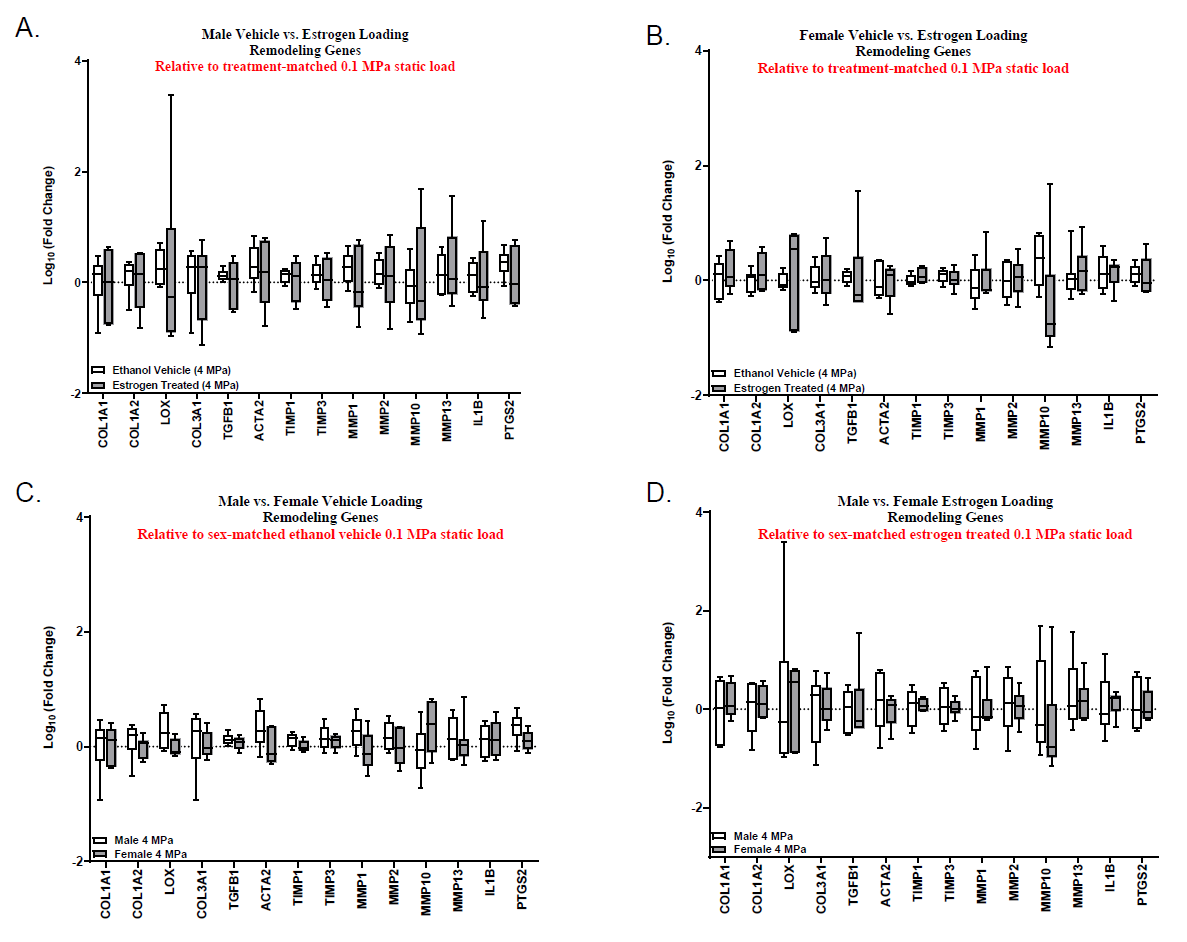


Supplementary Figure 3: **Comparisons of the male and female ACLs remodeling response to load.** RT-qPCR analysis of cyclically loaded male and female ACLs relative to their sex and treatment-matched 0.1 MPa static load. (A) Comparison of vehicle control and estrogen treatment in male samples. (B) Comparison of vehicle control and estrogen treatment in female samples. (C) Comparison of male and female ACLs response to vehicle treatment. (D) Comparison of male and female ACLs response to estrogen treatment (n=6 for all male samples, n=7 for female vehicle, n=6 for female estrogen treated). Data represented as box and whisker plot with the whiskers representing the min and max data.


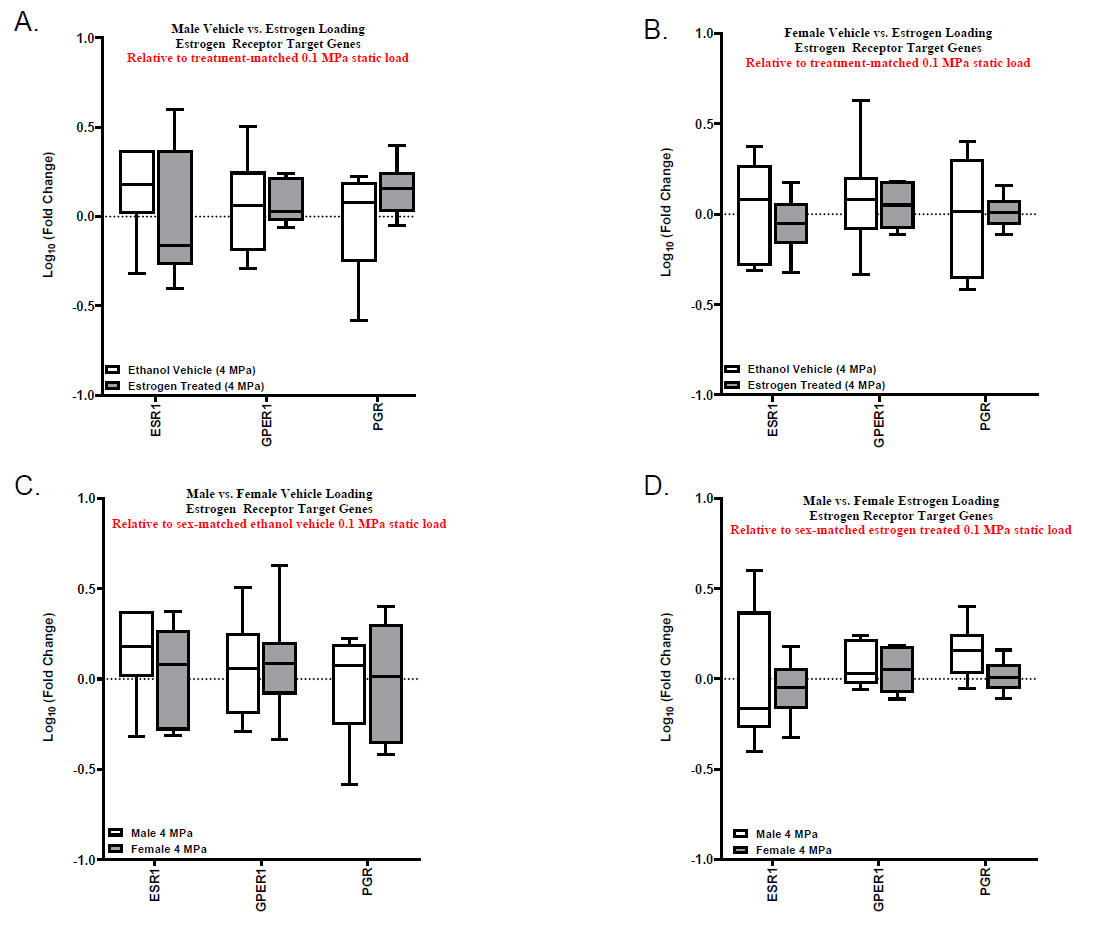


Supplementary Figure 4: **Comparisons of the male and female ACLs estrogen receptor target genes in response to load.** RT-qPCR analysis of cyclically loaded male and female ACLs relative to their sex and treatment-matched 0.1 MPa static load. (A) Comparison of vehicle control and estrogen treatment in male samples. (B) Comparison of vehicle control and estrogen treatment in female samples. (C) Comparison of male and female ACL response to vehicle treatment. (D) Comparison of male and female ACLs response to estrogen treatment (n=6 for all male samples, n=6 - 7 for female vehicle, n=6 for female estrogen treated). Data represented as box and whisker plot with the whiskers representing the min and max data.


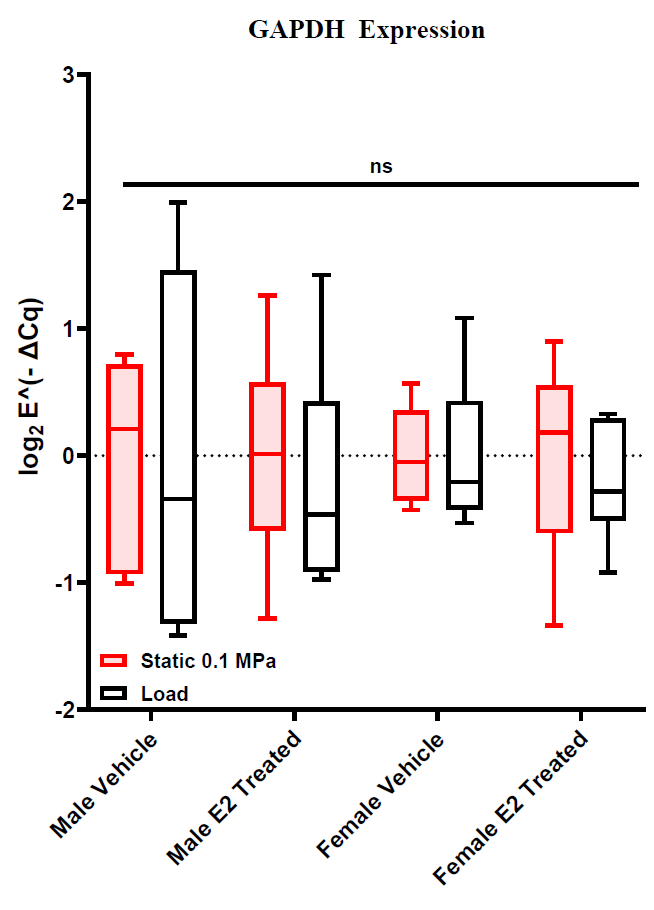


Supplementary Figure 5: **Comparison of housekeeping gene expression between groups.** RT-qPCR analysis of GAPDH for cyclically loaded male and female ACLs relative to their sex and treatment-matched 0.1 MPa static load (n=6 for all samples). Data represented as a box and whisker plot with the whiskers representing the min and max data.
