# Supplementary material for: Female Anterior Cruciate Ligaments Exhibit a Muted Mechanobiological Response to Mechanical Loading
Source: J Orthop Res. 2025 Oct 15;43(12):2188–202. doi: 10.1002/jor.70068 (PMC12604462; doi:10.1002/jor.70068)
Supplement: Supplementary file 2 — Supplementary Table 1: Summary of sample sizes for each experimental condition. Supplementary Table 2: List of Taqman probes and their efficiencies. Supplementary Table 3: List of differentially expressed genes in male ACL response to load. Supplementary Table 4: List of differentially expressed genes in female ACL response to load. Supplementary Table 5: List of differentially expressed genes in sex effect load (female ACL response to load compared to male ACL response to load). Supplementary Table 6: List of differentially expressed genes in sex effect static (statically loaded female ACLs relative to male statically loaded ACLs). Supplementary Table 7: List of differentially expressed genes in sex effect fresh (freshly harvested female ACLs relative to male freshly harvested ACLs). Supplementary Table 8: List of significantly enriched pathways in male ACL response to load. Pathways with a z‐score > 0 are predicted to be activated and z‐score < 0 are predicted to be inhibited. A prediction was unable to be made for pathways with a NA z‐Score. Supplementary Table 9: List of significantly enriched pathways in female ACL response to load. A prediction was unable to be made for pathways with a NA z‐Score. Supplementary Table 10: List of significantly enriched pathways in sex effect load (female ACL response to load compared to male ACL response to load). Pathways with a z‐score > 0 are predicted to be activated and z‐score < 0 are predicted to be inhibited. A prediction was unable to be made for pathways with a NA z‐Score. Supplementary Table 11: List of DEGs present in pathways associated with ECM organization in male ACL response to load and sex effect load. Supplementary Table 12: Sample sizes required based on post‐hoc power calculations conducted on the PCR remodeling genes assuming a power > 0.8 and type I error of 0.05. [file JOR-43-2188-s001.docx]

**Supplementary Table 1:** Summary of sample sizes for each experimental condition.

|  | **RNA-Seq** | | **RT-qPCR** | | | |
| --- | --- | --- | --- | --- | --- | --- |
| **Load** | **Male** | **Female** | **Male (Vehicle)** | **Female (Vehicle)** | **Male (E2 Treated)** | **Female (E2 Treated)** |
| **Static (0.1 MPa)** | 3 | 3 | 6 | 7 | 6 | 6 |
| **Cylic (4 MPa)** | 3 | 3 | 6 | 7 | 6 | 6 |
| **Fresh harvested** | 2 | 2 | 0 | 0 | 0 | 0 |

**Supplementary Table 2:** List of Taqman probes and their efficiencies.

| **Gene** | **Gene ID** | **Probe Efficiency** |
| --- | --- | --- |
| **COL1A1** | Oc03396073_g1 | 87% |
| **COL1A2** | Oc03396112_m1 | 79% |
| **LOX** | Oc03398373_m1 | 82% |
| **COL3A1** | Oc03398373_m1 | 84% |
| **TGFB1** | Oc04176122_u1 | 82% |
| **ACTA2** | Oc03399251_m1 | 86% |
| **TIMP1** | Oc03397606_m1 | 82% |
| **TIMP3** | Oc04096947_m1 | 83% |
| **MMP1** | Oc04250657_m1 | 91% |
| **MMP2** | Oc03397553_m1 | 83% |
| **MMP10** | Oc03396490_m1 | 90% |
| **MMP13** | Oc03396899_m1 | 90% |
| **IL1B** | Oc03823250_s1 | 85% |
| **PTGS2** | Oc03398295_m1 | 84% |
| **ESR1** | Oc06688292_m1 | 95% |
| **GPER** | Oc06675336_s1 | 83% |
| **PGR** | Oc03397748_m1 | 84% |
| **GAPDH** | Oc03823402_g1 | 81% |

**Supplementary Table 3:** List of differentially expressed genes in male ACL response to load.

| **Ensembl ID** | **Gene Symbol** | **Male Load logFC** | **Male Load adjpval** |
| --- | --- | --- | --- |
| ENSOCUG00000001085 | ALK | -6.79 | 0.041 |
| ENSOCUG00000022885 | COX6 | -6.30 | 0.012 |
| ENSOCUG00000007355 | DSG2 | -5.46 | 0.043 |
| ENSOCUG00000000049 | MASP1 | -5.34 | 0.026 |
| ENSOCUG00000012337 | COL21A1 | -5.17 | 0.020 |
| ENSOCUG00000009715 | NEK2 | -5.10 | 0.018 |
| ENSOCUG00000023797 |  | -5.06 | 0.016 |
| ENSOCUG00000026402 | IL2RA | -4.29 | 0.017 |
| ENSOCUG00000010888 | RSPO1 | -4.21 | 0.035 |
| ENSOCUG00000024481 | CAPN3 | -4.21 | 0.031 |
| ENSOCUG00000011350 | SPON1 | -4.15 | 0.020 |
| ENSOCUG00000015068 | KIF23 | -4.13 | 0.033 |
| ENSOCUG00000007052 | PENK | -4.08 | 0.050 |
| ENSOCUG00000010729 | RASL12 | -4.01 | 0.045 |
| ENSOCUG00000022649 | APLN | -4.00 | 0.040 |
| ENSOCUG00000022385 |  | -3.86 | 0.013 |
| ENSOCUG00000005133 |  | -3.86 | 0.025 |
| ENSOCUG00000008765 | CKAP2L | -3.79 | 0.040 |
| ENSOCUG00000014433 | NYAP2 | -3.78 | 0.032 |
| ENSOCUG00000011838 | CXCL5 | -3.67 | 0.040 |
| ENSOCUG00000006357 | ADAMTS12 | -3.67 | 0.010 |
| ENSOCUG00000016480 | GLIS1 | -3.60 | 0.036 |
| ENSOCUG00000008561 |  | -3.60 | 0.013 |
| ENSOCUG00000011482 | POSTN | -3.60 | 0.040 |
| ENSOCUG00000022014 | NOTCH3 | -3.60 | 0.025 |
| ENSOCUG00000026268 | ADIPOQ | -3.59 | 0.010 |
| ENSOCUG00000005356 | GPR158 | -3.57 | 0.041 |
| ENSOCUG00000022030 | BARHL1 | -3.52 | 0.040 |
| ENSOCUG00000009395 | CNTN1 | -3.52 | 0.020 |
| ENSOCUG00000015376 | S1PR3 | -3.40 | 0.050 |
| ENSOCUG00000027331 |  | -3.38 | 0.031 |
| ENSOCUG00000028094 | PRC1 | -3.34 | 0.016 |
| ENSOCUG00000000508 | OLFM3 | -3.33 | 0.040 |
| ENSOCUG00000002562 | PODN | -3.33 | 0.007 |
| ENSOCUG00000014689 | IGF1 | -3.32 | 0.030 |
| ENSOCUG00000004455 | GUCY1A1 | -3.26 | 0.044 |
| ENSOCUG00000014595 |  | -3.22 | 0.016 |
| ENSOCUG00000008272 | SRPX | -3.19 | 0.010 |
| ENSOCUG00000026917 |  | -3.18 | 0.024 |
| ENSOCUG00000021735 | ITGA7 | -3.11 | 0.014 |
| ENSOCUG00000014733 |  | -2.99 | 0.032 |
| ENSOCUG00000003877 | AHR | -2.98 | 0.001 |
| ENSOCUG00000006131 | SPARCL1 | -2.94 | 0.046 |
| ENSOCUG00000007922 | ST8SIA4 | -2.84 | 0.021 |
| ENSOCUG00000014857 | COL5A3 | -2.81 | 0.010 |
| ENSOCUG00000013152 | ATP8A1 | -2.81 | 0.043 |
| ENSOCUG00000024055 | MCAM | -2.79 | 0.040 |
| ENSOCUG00000009058 | HP | -2.78 | 0.043 |
| ENSOCUG00000012070 | SPNS2 | -2.76 | 0.043 |
| ENSOCUG00000016007 | NID1 | -2.74 | 0.016 |
| ENSOCUG00000001618 | HGF | -2.68 | 0.013 |
| ENSOCUG00000015574 | S1PR1 | -2.65 | 0.013 |
| ENSOCUG00000015220 | PECAM1 | -2.65 | 0.045 |
| ENSOCUG00000013232 | COL4A1 | -2.65 | 0.013 |
| ENSOCUG00000002522 | PTGFR | -2.64 | 0.049 |
| ENSOCUG00000016491 | KIF14 | -2.63 | 0.049 |
| ENSOCUG00000008771 | IL1A | -2.61 | 0.043 |
| ENSOCUG00000014083 | LZTS1 | -2.60 | 0.040 |
| ENSOCUG00000012217 | APCDD1 | -2.57 | 0.015 |
| ENSOCUG00000013049 | PLXND1 | -2.51 | 0.004 |
| ENSOCUG00000025048 | CXCR5 | -2.50 | 0.041 |
| ENSOCUG00000010449 | ADORA2B | -2.47 | 0.041 |
| ENSOCUG00000014657 | EFEMP1 | -2.46 | 0.013 |
| ENSOCUG00000003863 | CD74 | -2.45 | 0.039 |
| ENSOCUG00000017924 | MME | -2.40 | 0.028 |
| ENSOCUG00000017032 | GNG11 | -2.40 | 0.026 |
| ENSOCUG00000003801 | C1QTNF1 | -2.38 | 0.024 |
| ENSOCUG00000023359 | TINAGL1 | -2.35 | 0.036 |
| ENSOCUG00000013954 | CALCRL | -2.33 | 0.039 |
| ENSOCUG00000013276 | COL4A2 | -2.32 | 0.012 |
| ENSOCUG00000006784 | SH3PXD2B | -2.28 | 0.035 |
| ENSOCUG00000017542 |  | -2.25 | 0.049 |
| ENSOCUG00000013520 | NR2F2 | -2.23 | 0.014 |
| ENSOCUG00000015495 | PRDM1 | -2.21 | 0.041 |
| ENSOCUG00000007028 | MARCKS | -2.20 | 0.012 |
| ENSOCUG00000026922 | ADAMTS7 | -2.14 | 0.013 |
| ENSOCUG00000004579 | NRP1 | -2.10 | 0.040 |
| ENSOCUG00000003155 | PLAU | -2.09 | 0.002 |
| ENSOCUG00000014369 | GCNT2 | -2.07 | 0.047 |
| ENSOCUG00000027378 |  | -2.06 | 0.033 |
| ENSOCUG00000015713 | TMEM132A | -2.03 | 0.041 |
| ENSOCUG00000004928 | TGFBI | -2.02 | 0.010 |
| ENSOCUG00000008781 | ADAM12 | -2.00 | 0.049 |
| ENSOCUG00000024458 |  | -2.00 | 0.045 |
| ENSOCUG00000027251 | C1orf54 | -1.99 | 0.045 |
| ENSOCUG00000027603 |  | -1.94 | 0.050 |
| ENSOCUG00000010244 |  | -1.93 | 0.040 |
| ENSOCUG00000001814 | GUCY1B1 | -1.93 | 0.031 |
| ENSOCUG00000026383 | AKR1B10 | -1.93 | 0.013 |
| ENSOCUG00000009625 | ATP8B1 | -1.91 | 0.038 |
| ENSOCUG00000011670 | SEMA3F | -1.91 | 0.045 |
| ENSOCUG00000007506 | MARCHF3 | -1.90 | 0.041 |
| ENSOCUG00000003720 | DUSP4 | -1.88 | 0.031 |
| ENSOCUG00000008947 | MARCKSL1 | -1.86 | 0.020 |
| ENSOCUG00000013423 | STEAP3 | -1.84 | 0.043 |
| ENSOCUG00000008127 | CALD1 | -1.82 | 0.018 |
| ENSOCUG00000001336 | ARHGAP27 | -1.77 | 0.043 |
| ENSOCUG00000001429 | MAOA | -1.74 | 0.034 |
| ENSOCUG00000014676 | LAMB1 | -1.72 | 0.014 |
| ENSOCUG00000011060 | MYO1B | -1.71 | 0.010 |
| ENSOCUG00000014425 | FILIP1L | -1.71 | 0.040 |
| ENSOCUG00000005619 | HYAL1 | -1.69 | 0.041 |
| ENSOCUG00000021324 | NDP | -1.68 | 0.045 |
| ENSOCUG00000002203 | PTN | -1.68 | 0.043 |
| ENSOCUG00000010783 | STARD8 | -1.65 | 0.041 |
| ENSOCUG00000015145 | NFATC4 | -1.64 | 0.041 |
| ENSOCUG00000006714 | MMP2 | -1.62 | 0.028 |
| ENSOCUG00000025513 | IGFBP5 | -1.62 | 0.013 |
| ENSOCUG00000004900 | PALLD | -1.56 | 0.012 |
| ENSOCUG00000013643 | PDLIM1 | -1.51 | 0.014 |
| ENSOCUG00000005628 | JAM3 | -1.48 | 0.041 |
| ENSOCUG00000016507 | ATP2B1 | -1.47 | 0.025 |
| ENSOCUG00000009340 | ARAP3 | -1.46 | 0.039 |
| ENSOCUG00000008572 | RBPMS | -1.46 | 0.043 |
| ENSOCUG00000010005 | RAB8B | -1.46 | 0.013 |
| ENSOCUG00000010633 | HMOX1 | -1.41 | 0.021 |
| ENSOCUG00000013633 | DAB2IP | -1.41 | 0.023 |
| ENSOCUG00000011140 |  | -1.40 | 0.025 |
| ENSOCUG00000001030 | GEM | -1.38 | 0.026 |
| ENSOCUG00000008855 |  | -1.36 | 0.034 |
| ENSOCUG00000000978 | LOXL1 | -1.35 | 0.045 |
| ENSOCUG00000002299 | ENAH | -1.34 | 0.045 |
| ENSOCUG00000001168 | LUZP1 | -1.29 | 0.043 |
| ENSOCUG00000002874 | PTK7 | -1.28 | 0.045 |
| ENSOCUG00000024302 | LGALS1 | -1.27 | 0.030 |
| ENSOCUG00000010549 | MMD | -1.27 | 0.040 |
| ENSOCUG00000022141 | SLC35F6 | -1.25 | 0.013 |
| ENSOCUG00000003292 |  | -1.19 | 0.040 |
| ENSOCUG00000029299 | CHD3 | -1.18 | 0.040 |
| ENSOCUG00000015191 | EXT1 | -1.15 | 0.025 |
| ENSOCUG00000006565 | HYCC1 | -1.14 | 0.048 |
| ENSOCUG00000015389 | TEAD4 | -1.09 | 0.047 |
| ENSOCUG00000004406 | FBXL7 | -1.07 | 0.035 |
| ENSOCUG00000016023 | ADAM10 | -1.07 | 0.041 |
| ENSOCUG00000006086 | LCLAT1 | -1.04 | 0.040 |
| ENSOCUG00000017281 | B3GNT2 | -1.04 | 0.049 |
| ENSOCUG00000005211 | PDGFR | -1.00 | 0.034 |
| ENSOCUG00000023782 | IL11RA | 1.00 | 0.027 |
| ENSOCUG00000007324 | ADGRG2 | 1.02 | 0.033 |
| ENSOCUG00000008142 | TCF7L2 | 1.02 | 0.017 |
| ENSOCUG00000010558 | PCCB | 1.03 | 0.040 |
| ENSOCUG00000003144 | BPGM | 1.03 | 0.040 |
| ENSOCUG00000006059 | PFN2 | 1.03 | 0.030 |
| ENSOCUG00000008261 | NXT2 | 1.04 | 0.039 |
| ENSOCUG00000000889 |  | 1.04 | 0.043 |
| ENSOCUG00000001419 | CSAD | 1.04 | 0.041 |
| ENSOCUG00000016927 | SH3BGRL3 | 1.06 | 0.015 |
| ENSOCUG00000004694 | MINDY1 | 1.07 | 0.013 |
| ENSOCUG00000010740 | TARS3 | 1.08 | 0.041 |
| ENSOCUG00000015488 | COPRS | 1.08 | 0.030 |
| ENSOCUG00000003754 | RUNX1T1 | 1.08 | 0.041 |
| ENSOCUG00000017308 | FLOT1 | 1.09 | 0.012 |
| ENSOCUG00000010891 |  | 1.10 | 0.010 |
| ENSOCUG00000022348 | ZBTB7C | 1.12 | 0.040 |
| ENSOCUG00000008513 | MYO1D | 1.14 | 0.014 |
| ENSOCUG00000007344 | DSE | 1.14 | 0.044 |
| ENSOCUG00000009289 |  | 1.18 | 0.041 |
| ENSOCUG00000024700 |  | 1.18 | 0.026 |
| ENSOCUG00000015547 | LIMCH1 | 1.19 | 0.041 |
| ENSOCUG00000010834 | TBX4 | 1.21 | 0.022 |
| ENSOCUG00000017516 | RETREG1 | 1.21 | 0.050 |
| ENSOCUG00000007601 | MBNL1 | 1.22 | 0.039 |
| ENSOCUG00000017845 | MAB21L1 | 1.24 | 0.028 |
| ENSOCUG00000002320 | PIK3IP1 | 1.24 | 0.040 |
| ENSOCUG00000003377 | CYP27A1 | 1.26 | 0.021 |
| ENSOCUG00000015434 | MAP3K5 | 1.26 | 0.042 |
| ENSOCUG00000009306 | LARP6 | 1.29 | 0.018 |
| ENSOCUG00000026099 |  | 1.30 | 0.040 |
| ENSOCUG00000013799 | UACA | 1.31 | 0.042 |
| ENSOCUG00000003233 | TRIM7 | 1.32 | 0.040 |
| ENSOCUG00000025915 |  | 1.36 | 0.029 |
| ENSOCUG00000026941 | MEOX2 | 1.40 | 0.041 |
| ENSOCUG00000011934 | EPHX1 | 1.40 | 0.014 |
| ENSOCUG00000010384 | IL16 | 1.42 | 0.009 |
| ENSOCUG00000006974 |  | 1.43 | 0.045 |
| ENSOCUG00000021938 | PGER5 | 1.43 | 0.032 |
| ENSOCUG00000014659 | PALS2 | 1.46 | 0.050 |
| ENSOCUG00000003208 | DOCK8 | 1.48 | 0.016 |
| ENSOCUG00000012397 | TSC22D3 | 1.49 | 0.014 |
| ENSOCUG00000001214 |  | 1.49 | 0.011 |
| ENSOCUG00000002977 | RAB3IP | 1.50 | 0.035 |
| ENSOCUG00000010941 | CPXM2 | 1.51 | 0.013 |
| ENSOCUG00000003722 | ANGPT1 | 1.52 | 0.034 |
| ENSOCUG00000014245 | METRNL | 1.52 | 0.008 |
| ENSOCUG00000025358 | PROS1 | 1.54 | 0.029 |
| ENSOCUG00000007460 | SOD3 | 1.57 | 0.039 |
| ENSOCUG00000015564 | PLSCR4 | 1.58 | 0.013 |
| ENSOCUG00000017808 | NIM1K | 1.58 | 0.045 |
| ENSOCUG00000004390 | SHLD2 | 1.63 | 0.043 |
| ENSOCUG00000014660 | CNKSR2 | 1.63 | 0.041 |
| ENSOCUG00000002730 | LRRC1 | 1.65 | 0.026 |
| ENSOCUG00000026409 |  | 1.65 | 0.033 |
| ENSOCUG00000004753 | KANK1 | 1.65 | 0.016 |
| ENSOCUG00000025282 |  | 1.70 | 0.040 |
| ENSOCUG00000002887 | FRMD4B | 1.72 | 0.025 |
| ENSOCUG00000014903 |  | 1.74 | 0.032 |
| ENSOCUG00000022929 | DBNDD2 | 1.74 | 0.017 |
| ENSOCUG00000009045 |  | 1.75 | 0.014 |
| ENSOCUG00000016504 | ZNF704 | 1.75 | 0.047 |
| ENSOCUG00000023617 |  | 1.76 | 0.045 |
| ENSOCUG00000003521 | ANK3 | 1.78 | 0.031 |
| ENSOCUG00000012051 | ANKH | 1.81 | 0.014 |
| ENSOCUG00000017109 | TTC39C | 1.83 | 0.013 |
| ENSOCUG00000008484 | MAB21L2 | 1.85 | 0.009 |
| ENSOCUG00000012259 | CMKLR1 | 1.86 | 0.020 |
| ENSOCUG00000009707 | PRELP | 1.88 | 0.039 |
| ENSOCUG00000014063 |  | 1.93 | 0.045 |
| ENSOCUG00000006107 | GRAMD2A | 1.94 | 0.046 |
| ENSOCUG00000003513 |  | 2.00 | 0.037 |
| ENSOCUG00000010357 | MBOAT1 | 2.01 | 0.039 |
| ENSOCUG00000002244 | AMPH | 2.01 | 0.043 |
| ENSOCUG00000017890 | MDGA1 | 2.03 | 0.016 |
| ENSOCUG00000013354 | SLC1A6 | 2.05 | 0.037 |
| ENSOCUG00000011655 |  | 2.10 | 0.039 |
| ENSOCUG00000021767 | HYKK | 2.21 | 0.043 |
| ENSOCUG00000014223 | UVRAG | 2.24 | 0.010 |
| ENSOCUG00000007830 | IL31RA | 2.26 | 0.039 |
| ENSOCUG00000003498 | SPEF1 | 2.27 | 0.015 |
| ENSOCUG00000002660 | CALHM5 | 2.27 | 0.026 |
| ENSOCUG00000029200 | ANGPTL5 | 2.35 | 0.041 |
| ENSOCUG00000025160 | TCAP | 2.37 | 0.040 |
| ENSOCUG00000026303 |  | 2.38 | 0.012 |
| ENSOCUG00000017706 | ATL1 | 2.38 | 0.020 |
| ENSOCUG00000006542 | PROK2 | 2.39 | 0.041 |
| ENSOCUG00000027183 | NTN1 | 2.42 | 0.013 |
| ENSOCUG00000017622 | NOVA1 | 2.44 | 0.013 |
| ENSOCUG00000001801 | FOXP2 | 2.47 | 0.021 |
| ENSOCUG00000000271 | MAMDC2 | 2.52 | 0.013 |
| ENSOCUG00000003854 | TPMT | 2.52 | 0.041 |
| ENSOCUG00000012307 | PRXL2A | 2.54 | 0.007 |
| ENSOCUG00000014239 | TLCD4 | 2.61 | 0.030 |
| ENSOCUG00000010020 | DNAJC6 | 2.62 | 0.002 |
| ENSOCUG00000015746 | NTMT2 | 2.71 | 0.043 |
| ENSOCUG00000009890 | DPY19L2 | 2.75 | 0.047 |
| ENSOCUG00000002994 | CCDC3 | 2.83 | 0.009 |
| ENSOCUG00000002672 | ADAMTSL3 | 2.86 | 0.033 |
| ENSOCUG00000029534 |  | 2.87 | 0.014 |
| ENSOCUG00000006938 | CSDC2 | 2.93 | 0.041 |
| ENSOCUG00000011452 |  | 2.97 | 0.040 |
| ENSOCUG00000017764 | VEGFD | 2.99 | 0.013 |
| ENSOCUG00000012541 | SOSTDC1 | 3.04 | 0.020 |
| ENSOCUG00000017601 | CDH19 | 3.07 | 0.041 |
| ENSOCUG00000015248 | SHISA2 | 3.11 | 0.040 |
| ENSOCUG00000001360 | GAB3 | 3.15 | 0.022 |
| ENSOCUG00000025096 | SH3TC2 | 3.19 | 0.026 |
| ENSOCUG00000025111 | GALNT15 | 3.23 | 0.016 |
| ENSOCUG00000015957 | MYOC | 3.28 | 0.010 |
| ENSOCUG00000007364 | ELAPOR2 | 3.30 | 0.035 |
| ENSOCUG00000029257 |  | 3.46 | 0.039 |
| ENSOCUG00000004829 | ESR1 | 3.54 | 0.021 |
| ENSOCUG00000025065 |  | 3.59 | 0.037 |
| ENSOCUG00000013081 | ANGPTL7 | 3.62 | 0.014 |
| ENSOCUG00000000148 | CDO1 | 3.67 | 0.040 |
| ENSOCUG00000000732 | FGF9 | 3.80 | 0.025 |
| ENSOCUG00000008784 | NALCN | 3.96 | 0.009 |
| ENSOCUG00000016462 | IL1RL1 | 4.35 | 0.013 |
| ENSOCUG00000005375 | MANEAL | 4.40 | 0.041 |
| ENSOCUG00000028102 | NPS | 4.55 | 0.039 |
| ENSOCUG00000013331 |  | 5.81 | 0.015 |

**Supplementary Table 4:** List of differentially expressed genes in female ACL response to load.

| **Ensembl ID** | **Gene Symbol** | **Female Load logFC** | **Female Load adjpval** |
| --- | --- | --- | --- |
| ENSOCUG00000026115 |  | -5.94 | 0.044 |
| ENSOCUG00000013482 | CYTL1 | -3.36 | 0.050 |
| ENSOCUG00000004424 | MRPS33 | -1.58 | 0.050 |
| ENSOCUG00000006661 | TRIM47 | -1.57 | 0.044 |
| ENSOCUG00000027663 | H2AJ | -1.48 | 0.050 |
| ENSOCUG00000022123 | FXYD1 | -1.46 | 0.044 |
| ENSOCUG00000024282 | PLEKHB2 | 1.62 | 0.050 |
| ENSOCUG00000016848 |  | 2.15 | 0.044 |
| ENSOCUG00000014931 | GBP4 | 4.08 | 0.050 |
| ENSOCUG00000029275 |  | 4.56 | 0.012 |

**Supplementary Table 5:** List of differentially expressed genes in sex effect load (female ACL response to load compared to male ACL response to load).

| **Ensembl ID** | **Gene Symbol** | **Sex Effect Load logFC** | **Sex Effect Load adjpval** |
| --- | --- | --- | --- |
| ENSOCUG00000012541 | SOSTDC1 | -6.10 | 0.018 |
| ENSOCUG00000016462 | IL1RL1 | -5.62 | 0.015 |
| ENSOCUG00000008784 | NALCN | -5.56 | 0.013 |
| ENSOCUG00000000148 | CDO1 | -5.53 | 0.023 |
| ENSOCUG00000013482 | CYTL1 | -5.12 | 0.014 |
| ENSOCUG00000013081 | ANGPTL7 | -4.75 | 0.017 |
| ENSOCUG00000025111 | GALNT15 | -4.63 | 0.017 |
| ENSOCUG00000029534 |  | -4.12 | 0.017 |
| ENSOCUG00000002994 | CCDC3 | -3.96 | 0.014 |
| ENSOCUG00000015957 | MYOC | -3.84 | 0.016 |
| ENSOCUG00000002672 | ADAMTSL3 | -3.73 | 0.045 |
| ENSOCUG00000009707 | PRELP | -3.69 | 0.014 |
| ENSOCUG00000017622 | NOVA1 | -3.66 | 0.016 |
| ENSOCUG00000000271 | MAMDC2 | -3.60 | 0.014 |
| ENSOCUG00000007443 | RAB11FIP4 | -3.29 | 0.048 |
| ENSOCUG00000027183 | NTN1 | -3.25 | 0.017 |
| ENSOCUG00000002435 |  | -3.25 | 0.023 |
| ENSOCUG00000012307 | PRXL2A | -3.24 | 0.013 |
| ENSOCUG00000002887 | FRMD4B | -3.21 | 0.014 |
| ENSOCUG00000010020 | DNAJC6 | -3.07 | 0.013 |
| ENSOCUG00000026303 |  | -3.04 | 0.017 |
| ENSOCUG00000006107 | GRAMD2A | -2.96 | 0.042 |
| ENSOCUG00000012051 | ANKH | -2.72 | 0.014 |
| ENSOCUG00000010371 | FRZB | -2.69 | 0.035 |
| ENSOCUG00000009045 |  | -2.64 | 0.015 |
| ENSOCUG00000014903 |  | -2.56 | 0.041 |
| ENSOCUG00000017890 | MDGA1 | -2.39 | 0.047 |
| ENSOCUG00000022929 | DBNDD2 | -2.37 | 0.036 |
| ENSOCUG00000002977 | RAB3IP | -2.33 | 0.034 |
| ENSOCUG00000017845 | MAB21L1 | -2.28 | 0.017 |
| ENSOCUG00000014602 | ITGB5 | -2.27 | 0.018 |
| ENSOCUG00000007460 | SOD3 | -2.25 | 0.034 |
| ENSOCUG00000011195 | FZD9 | -2.24 | 0.036 |
| ENSOCUG00000003377 | CYP27A1 | -2.20 | 0.017 |
| ENSOCUG00000017109 | TTC39C | -2.19 | 0.037 |
| ENSOCUG00000015547 | LIMCH1 | -2.19 | 0.018 |
| ENSOCUG00000006661 | TRIM47 | -2.17 | 0.016 |
| ENSOCUG00000006225 | PROSER2 | -2.14 | 0.043 |
| ENSOCUG00000008645 | S100A4 | -2.12 | 0.018 |
| ENSOCUG00000013463 | MELTF | -2.11 | 0.023 |
| ENSOCUG00000015564 | PLSCR4 | -2.11 | 0.018 |
| ENSOCUG00000026099 |  | -2.05 | 0.024 |
| ENSOCUG00000021292 |  | -2.05 | 0.035 |
| ENSOCUG00000010384 | IL16 | -1.97 | 0.013 |
| ENSOCUG00000017516 | RETREG1 | -1.95 | 0.038 |
| ENSOCUG00000022007 | ISLR | -1.95 | 0.022 |
| ENSOCUG00000009616 | CRISPLD1 | -1.95 | 0.021 |
| ENSOCUG00000012397 | TSC22D3 | -1.93 | 0.020 |
| ENSOCUG00000016314 | TMEM119 | -1.92 | 0.042 |
| ENSOCUG00000011934 | EPHX1 | -1.92 | 0.018 |
| ENSOCUG00000004753 | KANK1 | -1.89 | 0.042 |
| ENSOCUG00000008484 | MAB21L2 | -1.89 | 0.026 |
| ENSOCUG00000005571 | CA5B | -1.88 | 0.047 |
| ENSOCUG00000004748 |  | -1.88 | 0.035 |
| ENSOCUG00000016927 | SH3BGRL3 | -1.88 | 0.013 |
| ENSOCUG00000009289 |  | -1.88 | 0.042 |
| ENSOCUG00000024700 |  | -1.88 | 0.019 |
| ENSOCUG00000010834 | TBX4 | -1.87 | 0.022 |
| ENSOCUG00000003144 | BPGM | -1.86 | 0.019 |
| ENSOCUG00000027663 | H2AJ | -1.84 | 0.035 |
| ENSOCUG00000010941 | CPXM2 | -1.80 | 0.029 |
| ENSOCUG00000022727 |  | -1.78 | 0.037 |
| ENSOCUG00000014245 | METRNL | -1.77 | 0.015 |
| ENSOCUG00000025135 |  | -1.76 | 0.048 |
| ENSOCUG00000000083 | HTRA4 | -1.75 | 0.043 |
| ENSOCUG00000007601 | MBNL1 | -1.72 | 0.035 |
| ENSOCUG00000010891 |  | -1.71 | 0.013 |
| ENSOCUG00000015488 | COPRS | -1.71 | 0.026 |
| ENSOCUG00000007324 | ADGRG2 | -1.67 | 0.021 |
| ENSOCUG00000026208 | ST6GALNAC6 | -1.67 | 0.035 |
| ENSOCUG00000006059 | PFN2 | -1.66 | 0.023 |
| ENSOCUG00000008513 | MYO1D | -1.65 | 0.016 |
| ENSOCUG00000012611 | NET1 | -1.65 | 0.017 |
| ENSOCUG00000008142 | TCF7L2 | -1.57 | 0.018 |
| ENSOCUG00000010376 | MGST3 | -1.55 | 0.041 |
| ENSOCUG00000017494 |  | -1.53 | 0.038 |
| ENSOCUG00000013444 |  | -1.52 | 0.042 |
| ENSOCUG00000012001 | GCDH | -1.51 | 0.048 |
| ENSOCUG00000023974 |  | -1.48 | 0.028 |
| ENSOCUG00000023782 | IL11RA | -1.48 | 0.028 |
| ENSOCUG00000006453 | NPR2 | -1.41 | 0.035 |
| ENSOCUG00000004694 | MINDY1 | -1.40 | 0.023 |
| ENSOCUG00000003620 | CCDC88A | -1.38 | 0.018 |
| ENSOCUG00000012223 | TXNDC15 | -1.32 | 0.018 |
| ENSOCUG00000023389 |  | -1.30 | 0.035 |
| ENSOCUG00000005929 | MRAS | -1.28 | 0.043 |
| ENSOCUG00000017308 | FLOT1 | -1.27 | 0.023 |
| ENSOCUG00000004114 | SLC25A37 | -1.25 | 0.043 |
| ENSOCUG00000017230 | PCCA | -1.23 | 0.044 |
| ENSOCUG00000029359 |  | -1.12 | 0.035 |
| ENSOCUG00000012726 |  | -1.11 | 0.035 |
| ENSOCUG00000000231 | AASDHPPT | 1.13 | 0.048 |
| ENSOCUG00000023809 | PICALM | 1.24 | 0.043 |
| ENSOCUG00000024010 | GPCPD1 | 1.34 | 0.048 |
| ENSOCUG00000007244 | PJA1 | 1.43 | 0.050 |
| ENSOCUG00000006086 | LCLAT1 | 1.50 | 0.036 |
| ENSOCUG00000011060 | MYO1B | 1.53 | 0.048 |
| ENSOCUG00000013049 | PLXND1 | 1.81 | 0.046 |
| ENSOCUG00000003155 | PLAU | 1.87 | 0.015 |
| ENSOCUG00000010005 | RAB8B | 1.87 | 0.019 |
| ENSOCUG00000024282 | PLEKHB2 | 2.06 | 0.028 |
| ENSOCUG00000009324 | GJA1 | 2.08 | 0.043 |
| ENSOCUG00000003877 | AHR | 2.29 | 0.014 |
| ENSOCUG00000014657 | EFEMP1 | 2.72 | 0.045 |
| ENSOCUG00000023286 |  | 2.73 | 0.026 |
| ENSOCUG00000015574 | S1PR1 | 2.74 | 0.035 |
| ENSOCUG00000014857 | COL5A3 | 2.91 | 0.028 |
| ENSOCUG00000002562 | PODN | 3.37 | 0.017 |
| ENSOCUG00000001618 | HGF | 3.50 | 0.018 |
| ENSOCUG00000006357 | ADAMTS12 | 3.55 | 0.035 |
| ENSOCUG00000029275 |  | 4.33 | 0.035 |
| ENSOCUG00000026547 | CADM4 | 4.38 | 0.048 |
| ENSOCUG00000000508 | OLFM3 | 4.76 | 0.026 |
| ENSOCUG00000014931 | GBP4 | 5.45 | 0.024 |
| ENSOCUG00000012337 | COL21A1 | 6.07 | 0.043 |
| ENSOCUG00000022030 | BARHL1 | 6.26 | 0.018 |

**Supplementary Table 6:** List of differentially expressed genes in sex effect static (statically loaded female ACLs relative to male statically loaded ACLs).

| **Ensembl ID** | **Gene Symbol** | **Sex Effect Static logFC** | **Sex Effect Static adjpval** |
| --- | --- | --- | --- |
| ENSOCUG00000007393 |  | -6.84 | 0.040 |
| ENSOCUG00000010888 | RSPO1 | -4.77 | 0.013 |
| ENSOCUG00000002114 | TRIM5 | -4.62 | 0.013 |
| ENSOCUG00000022030 | BARHL1 | -4.36 | 0.011 |
| ENSOCUG00000009455 | FRRS1 | -4.17 | 0.012 |
| ENSOCUG00000015068 | KIF23 | -4.04 | 0.032 |
| ENSOCUG00000012312 | CLEC4D | -3.79 | 0.025 |
| ENSOCUG00000005356 | GPR158 | -3.34 | 0.031 |
| ENSOCUG00000016480 | GLIS1 | -3.08 | 0.040 |
| ENSOCUG00000026402 | IL2RA | -2.56 | 0.019 |
| ENSOCUG00000001618 | HGF | -2.52 | 0.009 |
| ENSOCUG00000026917 |  | -2.36 | 0.040 |
| ENSOCUG00000003863 | CD74 | -2.10 | 0.043 |
| ENSOCUG00000029210 | AP5B1 | -1.82 | 0.031 |
| ENSOCUG00000026383 | AKR1B10 | -1.81 | 0.012 |
| ENSOCUG00000007028 | MARCKS | -1.59 | 0.013 |
| ENSOCUG00000003877 | AHR | -1.51 | 0.005 |
| ENSOCUG00000002562 | PODN | -1.50 | 0.047 |
| ENSOCUG00000024282 | PLEKHB2 | -1.28 | 0.037 |
| ENSOCUG00000011140 |  | -1.03 | 0.036 |
| ENSOCUG00000010005 | RAB8B | -1.03 | 0.040 |
| ENSOCUG00000004721 |  | 1.02 | 0.040 |
| ENSOCUG00000014245 | METRNL | 1.11 | 0.013 |
| ENSOCUG00000015547 | LIMCH1 | 1.16 | 0.050 |
| ENSOCUG00000001214 |  | 1.24 | 0.013 |
| ENSOCUG00000010384 | IL16 | 1.25 | 0.009 |
| ENSOCUG00000022690 |  | 1.39 | 0.037 |
| ENSOCUG00000012307 | PRXL2A | 1.61 | 0.037 |
| ENSOCUG00000009707 | PRELP | 1.63 | 0.040 |
| ENSOCUG00000015957 | MYOC | 1.74 | 0.040 |
| ENSOCUG00000026303 |  | 1.77 | 0.037 |
| ENSOCUG00000000271 | MAMDC2 | 1.79 | 0.033 |
| ENSOCUG00000014223 | UVRAG | 1.81 | 0.013 |
| ENSOCUG00000010371 | FRZB | 1.81 | 0.030 |
| ENSOCUG00000002994 | CCDC3 | 2.05 | 0.040 |
| ENSOCUG00000006107 | GRAMD2A | 2.23 | 0.040 |
| ENSOCUG00000006753 | OPTC | 3.70 | 0.036 |
| ENSOCUG00000016462 | IL1RL1 | 4.04 | 0.027 |
| ENSOCUG00000008784 | NALCN | 4.31 | 0.005 |
| ENSOCUG00000026115 |  | 6.96 | 0.004 |

**Supplementary Table 7:** List of differentially expressed genes in sex effect fresh (freshly harvested female ACLs relative to male freshly harvested ACLs).

| **Ensembl ID** | **Gene Symbol** | **Sex Effect Fresh logFC** | **Sex Effect Fresh adjpval** |
| --- | --- | --- | --- |
| ENSOCUG00000002203 | PTN | -2.51 | 0.020 |
| ENSOCUG00000026333 | PER1 | 1.64 | 0.046 |

**Supplementary Table 8:** List of significantly enriched pathways in male ACL response to load. Pathways with a z-score > 0 are predicted to be activated and z-score < 0 are predicted to be inhibited. A prediction was unable to be made for pathways with a NA z-Score.

| **Pathway** | **Zscore** |
| --- | --- |
| Regulation of Insulin-like Growth Factor (IGF) transport and uptake by IGFBPs | -3 |
| Post-translational protein phosphorylation | -3 |
| Collagen degradation | -2 |
| Docosahexaenoic Acid (DHA) Signaling | -2 |
| Molecular Mechanisms of Cancer | -2 |
| Degradation of the extracellular matrix | -2 |
| Integrin cell surface interactions | -2 |
| Platelet homeostasis | -2 |
| Extracellular matrix organization | -2 |
| Semaphorin Neuronal Repulsive Signaling Pathway | -2 |
| Pulmonary Fibrosis Idiopathic Signaling Pathway | -2 |
| Collagen chain trimerization | -2 |
| NCAM signaling for neurite out-growth | -2 |
| Collagen biosynthesis and modifying enzymes | -2 |
| Breast Cancer Regulation by Stathmin1 | -2 |
| CREB Signaling in Neurons | -2 |
| BBSome Signaling Pathway | -2 |
| Interleukin-4 and Interleukin-13 signaling | -2 |
| GP6 Signaling Pathway | -2 |
| HIF1α Signaling | -2 |
| Role of Osteoblasts in Rheumatoid Arthritis Signaling Pathway | -2 |
| G-Protein Coupled Receptor Signaling | -2 |
| Inhibition of Matrix Metalloproteases | 2 |
| Ephrin A Signaling | 2 |
| Role of Osteoclasts in Rheumatoid Arthritis Signaling Pathway | -1 |
| Cachexia Signaling Pathway | -1 |
| Pathogen Induced Cytokine Storm Signaling Pathway | -1 |
| IL-10 Signaling | -1 |
| Adrenomedullin signaling pathway | -1 |
| Multiple Sclerosis Signaling Pathway | -1 |
| S100 Family Signaling Pathway | -1 |
| Class A/1 (Rhodopsin-like receptors) | -1 |
| Lung Ionic Balance Signaling Pathway | -1 |
| RHO GTPase cycle | -1 |
| Tumor Microenvironment Pathway | -1 |
| Eicosanoid Signaling | -1 |
| STAT3 Pathway | -1 |
| Extra-nuclear estrogen signaling | -1 |
| FAK Signaling | -1 |
| cAMP-mediated signaling | -1 |
| Serotonin Receptor Signaling | -1 |
| L1CAM interactions | -1 |
| Cardiac Hypertrophy Signaling | -1 |
| G alpha (s) signalling events | -1 |
| Epithelial Adherens Junction Signaling | -1 |
| HEY1 Signaling Pathway | -1 |
| Mitochondrial Division Signaling Pathway | -1 |
| O-linked glycosylation | -1 |
| Role of Chondrocytes in Rheumatoid Arthritis Signaling Pathway | -1 |
| WNT/SHH Axonal Guidance Signaling Pathway | -1 |
| Preeclampsia Signaling Pathway | -1 |
| Pulmonary Healing Signaling Pathway | -1 |
| Estrogen Receptor Signaling | -1 |
| Irritable Bowel Syndrome Signaling Pathway | -1 |
| Wound Healing Signaling Pathway | -1 |
| Sheddase Signaling Pathway | -1 |
| Sertoli Cell-Sertoli Cell Junction Signaling | 1 |
| Hematoma Resolution Signaling Pathway | 1 |
| PIP3 activates AKT signaling | 1 |
| Osteoarthritis Pathway | 1 |
| Glycosaminoglycan metabolism | 0 |
| Response to elevated platelet cytosolic Ca2+ | 0 |
| Bone Mineralization Signaling Pathway | 0 |
| Colorectal Cancer Metastasis Signaling | 0 |
| Ion channel transport | 0 |
| IL-8 Signaling | 0 |
| Cell surface interactions at the vascular wall | 0 |
| Hepatic Cholestasis | 0 |
| RAF/MAP kinase cascade | 0 |
| Cardiac Hypertrophy Signaling (Enhanced) | 0 |
| Sertoli Cell-Germ Cell Junction Signaling Pathway (Enhanced) | 0 |
| BEX2 Signaling Pathway | 0 |
| Phase I - Functionalization of compounds | 0 |
| Hereditary Breast Cancer Signaling | 0 |
| Th2 Pathway | 0 |
| eNOS Signaling | 0 |
| Protein Kinase A Signaling | 0 |
| HOTAIR Regulatory Pathway | 0 |
| Cellular Effects of Sildenafil (Viagra) | 0 |
| ROBO SLIT Signaling Pathway | 0 |
| Glycation Signaling Pathway | 0 |
| Hepatic Fibrosis / Hepatic Stellate Cell Activation | NA |
| Axonal Guidance Signaling | NA |
| Taurine Biosynthesis | NA |
| Granulocyte Adhesion and Diapedesis | NA |
| Signaling by ALK | NA |
| Role of Osteoblasts, Osteoclasts and Chondrocytes in Rheumatoid Arthritis | NA |
| Smooth Muscle Contraction | NA |
| Apelin Endothelial Signaling Pathway | NA |
| Gαi Signaling | NA |
| Endocannabinoid Cancer Inhibition Pathway | NA |
| Signaling by NOTCH3 | NA |
| IL-1 Signaling | NA |
| Microautophagy Signaling Pathway | NA |
| Glucocorticoid Receptor Signaling | NA |
| Agranulocyte Adhesion and Diapedesis | NA |
| RHO GTPases activate CIT | NA |
| Signaling by PDGF | NA |
| Assembly of collagen fibrils and other multimeric structures | NA |
| Th1 and Th2 Activation Pathway | NA |
| NFE2L2 regulating anti-oxidant/detoxification enzymes | NA |
| Bladder Cancer Signaling | NA |
| Sphingosine-1-phosphate Signaling | NA |
| Interleukin-6 family signaling | NA |
| Gαs Signaling | NA |
| GNRH Signaling | NA |
| Dopamine Receptor Signaling | NA |
| Regulation of the Epithelial-Mesenchymal Transition Pathway | NA |
| Sulfur amino acid metabolism | NA |
| CDP-diacylglycerol Biosynthesis I | NA |
| EGR2 and SOX10-mediated initiation of Schwann cell myelination | NA |
| PI3K/AKT Signaling | NA |
| Tryptophan Degradation X (Mammalian, via Tryptamine) | NA |
| Phosphatidylglycerol Biosynthesis II (Non-plastidic) | NA |
| Antiproliferative Role of Somatostatin Receptor 2 | NA |
| Thyroid Cancer Signaling | NA |
| Signaling by MET | NA |
| Role of JAK family kinases in IL-6-type Cytokine Signaling | NA |
| Activation of Matrix Metalloproteinases | NA |
| Inhibition of Angiogenesis by TSP1 | NA |
| Signaling by NOTCH2 | NA |
| Heme Degradation | NA |
| Methylmalonyl Pathway | NA |
| Rapoport-Luebering Glycolytic Shunt | NA |
| L-cysteine Degradation I | NA |
| Oxytocin in Spinal Neurons Signaling Pathway | NA |
| Coagulation System | NA |
| FGF Signaling | NA |
| ABRA Signaling Pathway | NA |
| Superpathway of Methionine Degradation | NA |
| Tuberculosis Latent Signaling Pathway | NA |
| Notch Signaling | NA |
| Role of NFAT in Cardiac Hypertrophy | NA |
| 2-oxobutanoate Degradation I | NA |
| Melatonin Degradation II | NA |
| Ovarian Cancer Signaling | NA |
| Apelin Adipocyte Signaling Pathway | NA |
| Neurotransmitter release cycle | NA |
| Intrinsic Prothrombin Activation Pathway | NA |

**Supplementary Table 9:** List of significantly enriched pathways in female ACL response to load. A prediction was unable to be made for pathways with a NA z-Score.

| **Pathway** | **Zscore** |
| --- | --- |
| DNA methylation | NA |
| Activated PKN1 stimulates transcription of AR regulated genes KLK2 and KLK3 | NA |
| SIRT1 negatively regulates rRNA expression | NA |
| Chromatin modifications during the maternal to zygotic transition (MZT) | NA |
| PRC2 methylates histones and DNA | NA |
| ERCC6 (CSB) and EHMT2 (G9a) positively regulate rRNA expression | NA |
| Nucleosome assembly | NA |
| Meiotic recombination | NA |
| Transcriptional regulation of granulopoiesis | NA |
| B-WICH complex positively regulates rRNA expression | NA |
| Meiotic synapsis | NA |
| DNA Damage/Telomere Stress Induced Senescence | NA |
| RUNX1 regulates megakaryocyte differentiation and platelet function | NA |
| NoRC negatively regulates rRNA expression | NA |
| Senescence-Associated Secretory Phenotype (SASP) | NA |
| RNA Polymerase I Transcription | NA |
| Pre-NOTCH Expression and Processing | NA |
| Activation of anterior HOX genes in hindbrain during early embryogenesis | NA |
| Oxidative Stress Induced Senescence | NA |
| Telomere Maintenance | NA |
| Mitochondrial translation | NA |
| Gene Silencing by RNA | NA |
| Mitotic Prophase | NA |
| DNA Replication Pre-Initiation | NA |
| Regulation of endogenous retroelements | NA |
| ESR-mediated signaling | NA |
| Cardiac conduction | NA |
| Transcriptional regulation by RUNX1 | NA |
| Ion channel transport | NA |
| TCF dependent signaling in response to WNT | NA |
| Chromatin organization | NA |

**Supplementary Table 10:** List of significantly enriched pathways in sex effect load (female ACL response to load compared to male ACL response to load). Pathways with a z-score > 0 are predicted to be activated and z-score < 0 are predicted to be inhibited. A prediction was unable to be made for pathways with a NA z-Score.

| **Pathway** | **Zscore** |
| --- | --- |
| Hepatic Fibrosis Signaling Pathway | -2 |
| Myelination Signaling Pathway | -2 |
| FAK Signaling | -2 |
| Autism Signaling Pathway | -2 |
| Molecular Mechanisms of Cancer | -1 |
| Osteoarthritis Pathway | -1 |
| Gap Junction Signaling | -1 |
| Role of Macrophages, Fibroblasts and Endothelial Cells in Rheumatoid Arthritis | -1 |
| Pulmonary Fibrosis Idiopathic Signaling Pathway | 0 |
| WNT/β-catenin Signaling | 0 |
| RHO GTPase cycle | 0 |
| Sertoli Cell-Sertoli Cell Junction Signaling | 0 |
| Wound Healing Signaling Pathway | 0 |
| Role of Osteoclasts in Rheumatoid Arthritis Signaling Pathway | 0 |
| STAT3 Pathway | NA |
| Axonal Guidance Signaling | NA |
| Ovarian Cancer Signaling | NA |
| Macropinocytosis Signaling | NA |
| NRF2-mediated Oxidative Stress Response | NA |
| Acyl Carrier Protein Metabolism | NA |
| Syndecan interactions | NA |
| Hepatic Fibrosis / Hepatic Stellate Cell Activation | NA |
| Regulation of the Epithelial-Mesenchymal Transition Pathway | NA |
| PI3K/AKT Signaling | NA |
| Mouse Embryonic Stem Cell Pluripotency | NA |
| Phase I - Functionalization of compounds | NA |
| O-linked glycosylation | NA |
| Superpathway of Methionine Degradation | NA |
| Role of Osteoblasts, Osteoclasts and Chondrocytes in Rheumatoid Arthritis | NA |
| Oncostatin M Signaling | NA |
| Elastic fibre formation | NA |
| Collagen chain trimerization | NA |
| HGF Signaling | NA |
| Methylmalonyl Pathway | NA |
| Rapoport-Luebering Glycolytic Shunt | NA |
| L-cysteine Degradation I | NA |
| Bone Mineralization Signaling Pathway | NA |
| Taurine Biosynthesis | NA |
| Lysine Degradation II | NA |
| Lysine Degradation V | NA |
| 2-oxobutanoate Degradation I | NA |
| Epithelial Adherens Junction Signaling | NA |
| Microautophagy Signaling Pathway | NA |
| PCP (Planar Cell Polarity) Pathway | NA |
| Glucocorticoid Receptor Signaling | NA |
| Xenobiotic Metabolism General Signaling Pathway | NA |
| Mitochondrial Division Signaling Pathway | NA |
| Collagen biosynthesis and modifying enzymes | NA |
| Aryl hydrocarbon receptor signalling | NA |
| Tumor Microenvironment Pathway | NA |
| Basal Cell Carcinoma Signaling | NA |
| Glioma Invasiveness Signaling | NA |
| Superoxide Radicals Degradation | NA |
| trans-Golgi Network Vesicle Budding | NA |
| Xenobiotic Metabolism Signaling | NA |
| Antiproliferative Role of Somatostatin Receptor 2 | NA |
| Renal Cell Carcinoma Signaling | NA |
| Thyroid Cancer Signaling | NA |
| Role of WNT/GSK-3β Signaling in the Pathogenesis of Influenza | NA |
| Signaling by MET | NA |
| Caveolar-mediated Endocytosis Signaling | NA |
| PPARα/RXRα Activation | NA |
| Metabolism of water-soluble vitamins and cofactors | NA |
| Pulmonary Healing Signaling Pathway | NA |
| Sheddase Signaling Pathway | NA |
| PEDF Signaling | NA |
| Integrin cell surface interactions | NA |
| Regulation of the Epithelial Mesenchymal Transition in Development Pathway | NA |
| Integrin Signaling | NA |
| Regulation of Cellular Mechanics by Calpain Protease | NA |
| RAB GEFs exchange GTP for GDP on RABs | NA |
| BMP signaling pathway | NA |
| Cardiac Hypertrophy Signaling (Enhanced) | NA |
| Ceramide Signaling | NA |
| Actin Nucleation by ARP-WASP Complex | NA |
| Reversible hydration of carbon dioxide | NA |
| Lysine catabolism | NA |
| Acute Myeloid Leukemia Signaling | NA |
| Post-translational modification: synthesis of GPI-anchored proteins | NA |
| Mitochondrial iron-sulfur cluster biogenesis | NA |
| Dissolution of Fibrin Clot | NA |
| Sertoli Cell-Germ Cell Junction Signaling Pathway (Enhanced) | NA |

**Supplementary Table 11:** List of DEGs present in pathways associated with ECM organization in male ACL response to load and sex effect load.

| **Male Load** | | **Sex Effect Load** | |
| --- | --- | --- | --- |
| **Pathway** | **DEGs in Pathway** | **Pathway** | **DEGs in Pathway** |
| Collagen degradation | ADAM10,COL4A1,COL4A2,COL5A3,MMP14,MMP2 | Elastic fibre formation | EFEMP1,ITGB5 |
| Inhibition of Matrix Metalloproteases | ADAM10,ADAM12,MMP14,MMP2 | Collagen chain trimerization | COL21A1,COL5A3 |
| Degradation of the extracellular matrix | ADAM10,CAPN3,LAMB1,MMP14,MMP2 | Collagen biosynthesis and modifying enzymes | COL21A1,COL5A3 |
| Extracellular matrix organization | ADAM12,COL4A1,COL4A2,COL5A3,LAMB1 |  |  |
| Collagen biosynthesis and modifying enzymes | COL21A1,COL4A1,COL4A2,COL5A3 |  |  |
| Activation of Matrix Metalloproteinases | MMP14,MMP2 |  |  |
| Assembly of collagen fibrils and other multimeric structures | COL4A1,COL4A2,COL5A3 |  |  |

**Supplementary Table 12:** Sample sizes required based on post-hoc power calculations conducted on the PCR remodeling genes assuming a power > 0.8 and type I error of 0.05.

| **Gene** | **DMSO Vehicle Control** | **Estrogen Treated** |
| --- | --- | --- |
| COL1A1 | 29 | 1235 |
| COL1A2 | 9 | 4526 |
| LOX | 1879 | 24 |
| COL3A1 | 20 | 99140 |
| TGFB | 65 | 4404 |
| ACTA | 25 | 170 |
| TIMP1 | 176 | 1235 |
| TIMP3 | 66 | 577 |
| MMP1 | 162 | 85989 |
| MMP2 | 221 | 475 |
| MMP10 | 156 | 8177 |
| MMP13 | 74877 | 143 |
| IL1B | 29 | 1035 |
| PTGS2 | 61 | 478 |
